# Docosahexaenoic acid supplementation inhibits monocyte exhaustion memory formation during sepsis

**DOI:** 10.1007/s00011-026-02194-w

**Published:** 2026-02-18

**Authors:** Blake A. Caldwell, Yajun Wu, Susanti Ie, Amy Lucas, Benjamin Conacher, Yao Zhang, Babak Razani, Liwu Li

**Affiliations:** 1 Department of Biological Sciences, Virginia Tech, Blacksburg, VA 24061-0910 USA; 2 Carillion Roanoke Memorial Hospital, Roanoke, VA 24014 USA; 3 Genetics, Bioinformatics, and Computational Biology Program, Virginia Tech, Blacksburg, VA 24061 USA; 4 Department of Medicine and Vascular Medicine Institute, University of Pittsburgh School of Medicine and UPMC, Pittsburgh, PA 15261 USA

**Keywords:** Sepsis, Docosahexaenoic acid (DHA), Monocyte exhaustion, Innate immune memory, DNA methylation

## Abstract

**Objective and design:**

Docosahexaenoic acid (DHA) is an omega-3 fatty acid with important roles in inflammation resolution. We tested the impact of DHA supplementation on monocyte exhaustion, an immune memory state contributing to chronic inflammation and immunosuppression following sepsis.

**Materials or subjects:**

Ex vivo sepsis modeling was performed with C57BL/6 mouse bone marrow monocytes (BMMCs) and peripheral blood mononuclear cells (PBMCs) from septic patients.

**Treatment:**

BMMCs stimulated with lipopolysaccharide (100 ng/mL) for 5 days were supplemented with 60 µM DHA. Septic patient PBMCs were treated for 24 h with 0, 15, 30, 45, or 60 µM DHA.

**Methods:**

Monocyte exhaustion was assayed by flow cytometry, qRT-PCR, and cytometric arrays. DNA methylation changes linked to exhaustion memory were measured by bisulfite pyrosequencing. Western blots were performed to link DHA treatment to altered cell signaling pathways in septic monocytes.

**Results:**

DHA supplementation suppresses the expression major exhaustion regulators CD38 and PD-L1 and dampens inflammatory cytokine transcription. These effects were mechanistically linked to STAT1/3 inhibition and accompanied by altered DNA methylation at immune regulators. DHA treatment also reduced CD157 cell surface levels and CCL2 secretion, both contributors to tissue invasion and injury during sepsis.

**Conclusions:**

Our results support the therapeutic application of DHA for the prevention of chronic immune dysfunction in sepsis survivors.

**Supplementary Information:**

The online version contains supplementary material available at 10.1007/s00011-026-02194-w.

## Introduction

Sepsis is responsible for 1 in 5 deaths worldwide and remains one of the most expensive medical disorders to treat [1, 2]. During sepsis, dysregulation of the body’s natural immune response leads to systemic organ and tissue damage. Improvements in patient care have shifted the clinical burden of sepsis from this early inflammatory phase to chronic immune dysfunction in survivors, with complications from persistent inflammation, immunosuppression, and catabolism syndrome (PICS) accounting for 75% of sepsis-related deaths [3–6]. Innate immune dysregulation is thought to be a major contributor to sepsis disease progression [7, 8]. In particular, monocytes develop a state of chronic immune “exhaustion” characterized by paradoxical pro-inflammatory and immunosuppressive activity coupled with reduced immune effector functions [9–12]. Long-term immune memory in monocytes is driven by epigenetic alterations in the form of histone modifications, chromatin remodeling, and DNA methylation [13–15]. Resolution of this pathogenic immune memory remains a major translational target for the treatment of PICS [16]. 

Docosahexaenoic acid (DHA) is an omega-3 fatty acid with potent immune regulatory effects. Following an acute inflammatory event, DHA is actively converted into specialized pro-resolving mediator (SPM) lipid signaling molecules that suppress neutrophil influx and inflammation and promote efferocytosis via monocyte and macrophage recruitment [17]. Monocytic lineage cells themselves can synthesize maresin-class SPMs from DHA, promoting a class switch from pro-inflammatory M1 to pro-resolving M2 macrophages via suppressed interferon signaling and inhibiting the synthesis of pro-inflammatory arachidonic acid derivatives [18–21]. Independent of its role as an SPM precursor, DHA can also impact immune cell behavior through either: (1) Direct incorporation into cell membranes, disrupting the organization of immune signaling molecules via altered membrane fluidity and lipid raft organization, or (2) Metabolism into various oxidized intermediates that act as PPARα/γ agonists or exhibit their own pro-resolving activity [22–24]. In the context of sepsis, omega-3 derivatives have a protective effect, enhancing bacterial clearance, suppressing pro-inflammatory cytokine secretion, and limiting tissue invasion and injury by innate cells [25–28]. While omega-3 administration has not been shown to impact sepsis patient mortality in clinical trials, it can reduce the length of ICU stays and limit the risk of secondary infection [29, 30]. Despite these promising results, the effect of early DHA intervention on chronic immune dysfunction after sepsis remains unexplored.

We hypothesized that DHA supplementation could interfere in the establishment of pathogenic innate immune memory, ameliorating the chronic immune dysregulation observed in PICS. In a mouse ex vivo sepsis model, we found that DHA supplementation inhibited the development of monocyte exhaustion at both the epigenetic and transcriptional levels. DHA-treated septic monocytes exhibit reduced mitochondrial disruption and improved survival via STAT1/3 inhibition. Similar effects were observed in the short-term treatment of sepsis patient peripheral blood mononuclear cell (PBMC) culture, notably with DHA reducing CD157 expression and the secretion of chemoattractant CCL2. Taken together, these results support the therapeutic application of DHA to combat pathogenic innate immune memory states following septic injury.

## Materials and methods

### Animal husbandry

Wild-type (WT) C57BL/6 mice were purchased from The Jackson Laboratory and regularly maintained in our laboratory. Mice were housed in a pathogen-free facility with 12–12 h light-dark cycles and free access to water and standard chow. All experimental analyses were conducted on 6- to 8-week-old female mice. Experiments with mice were approved by the Institutional Animal Care and Use Committee (IACUC) of Virginia Tech in accordance with the U.S. National Institutes of Health Guide for the Care and Use of Laboratory Animals.

### Clinical samples and inclusion criteria

Blood draws were collected from septic patients two days after ICU entry (Table S1). The study inclusion criteria are adult patients 18 years of age or older suspected to be septic by either: (1) Fulfilling systemic inflammatory response syndrome (SIRS) criteria, which includes heart rate > 90 bpm, respiratory rate > 20 breaths/min, WBC > 12,000 or < 4000, bands > 10%, and temperature > 100.9 °F or < 96.8 °F, or (2) Having a positive quick Sequential Organ Failure Assessment (qSOFA). A qSOFA score is considered positive if 2 or more of the following criteria are met: respiratory rate ≥ 2 breaths/min, systolic blood pressure ≤ 100 mmHg, or Glasgow Coma Score ≤ 13. Exclusion criteria for this study include patients with a presenting hemoglobin of < 9.0 mg/dL, evidence of active bleeding, concern for hemorrhagic shock or having received blood transfusion within the past 90 days, patients with an active or past medical history of white blood cell disorders, patients whose first set of blood cultures were drawn over 24 h prior to enrollment, pregnant patients, and patients who are currently imprisoned or incarcerated. Patient blood cultures were drawn within 24 h of inclusion. Blood samples were processed through erythrocyte sedimentation in 3% dextran and followed by ACK lysis (Quality Biological). Residual leukocytes were applied to PBMC culture. All experiments were approved by a joint Institutional Review Board through Virginia Tech and Carillion Roanoke Memorial Hospital (protocol 20–823). Samples were collected with the written informed consent of all participants or their legal guardian. It was conducted and performed in compliance with the ethical standards set out in the Declaration of Helsinki. Investigators were blinded to all patient information until the completion of the study.

### Cell culture

Mouse ex vivo BMMC LPS exhaustion experiments were performed as previously described [12, 31]. Briefly, bone marrow cells were harvested from 6- to 8-week-old C57BL/6 female mice, seeded at a density of 3 × 10^5^ cells/cm^2^, and cultured in complete RPMI 1640 media [10% fetal bovine serum (FBS), 1% penicillin-streptomycin, 1% L-glutamine] supplemented with 10 ng/mL mouse M-CSF (PeproTech). To eliminate confounding effects of variable endogenous DHA concentrations across FBS batches, frozen aliquots from a single FBS stock (TCB) were used across all experiments. Cells were cultured for 5 days at 37 °C in a humidified 5% CO_2_ atmosphere under continuous high-dose 100 ng/mL lipopolysaccharide (LPS; Sigma-Aldrich) stimulation or PBS control conditions, with fresh media changes at days 2 and 4 of culture, as previously described [31]. For DHA treatment, complete RPMI 1640 media was pre-mixed with cis-4,7,10,13,16,19-docosahexaenoic acid (DHA; Sigma-Aldrich) suspended in DMSO (0.1% DMSO final concentration) before application to cultured cells. Based on previously reported physiological DHA concentrations and dose-response analysis, 60 µM DHA was selected for mouse BMMC culture (Fig. S1) [32]. For mTOR inhibition experiments, BMMCs were cultured for the full 5 day duration in the presence of AZD2014 (Selleckchem; 0.5µM optimized dose) [33]. Unless otherwise stated, cells were supplemented with DHA for the full 5-day duration of BMMC culture. For human PBMC culture, following erythrocyte sedimentation and lysis, septic patient leukocytes were cultured at a density of 1 × 10^6^ cells/mL in complete RPMI 1640 medium supplemented with human M-CSF (10 ng/mL; PeproTech), as previously described. Cells were cultured in the presence of DMSO (0.1%) or 15, 30, 45, or 60µM DHA for 24 h at 37 °C in a humidified atmosphere with 5% CO_2_.

### Flow cytometry

Cultured mouse BMMCs were washed with PBS, harvested, and blocked in 1:100 Fc block (BD Biosciences) for 20 min at 4 °C. Cells were then incubated for 30 min at 4 °C with the following monocyte exhaustion antibody panel: Ly6C (RRID: AB_3685063, clone HK1.4), CD11b (AB_2738184, M1/70), CXCR2 (AB_2750073, SA044G4), CX3CR1 (AB_2565700, SA011F11) F4/80 (AB_2894417, BM8), CD38 (AB_2871855, 90/CD38), PD-L1 (AB_2738911, MIH5), MARCO (AB_3648917, 2359 A), CD157 (AB_10642685, BP-3), CD86 (AB_493343, GL-1), CD200R (AB_2244385, OX-110), CD74 (AB_2871143, In-1), and MHCII (AB_2191073, M5/114.15.2). BMMCs were then washed with FACS buffer (HBSS with 2% fetal bovine serum, FBS) and resuspended in FACS buffer containing propidium iodide (Invitrogen) for flow cytometry analysis. For mTOR inhibition experiments, BMMCs were processed for flow cytometry as described above and incubated with the following panel: Ly6C (AB_1732082, HK1.4), CD11b (AB_830642, M1/70), CD86 (AB_313149, GL-1), PD-L1 (AB_10612741, 10 F.9G2), and CD38 (AB_312929, 90). To detect BMMC mitochondrial membrane potentials, cells were stained using a MitoTracker Deep Red FM kit (Invitrogen) as described by the manufacturer’s instructions. To measure cell viability, BMMCs were labeled with an Annexin V Apoptosis Detection Kit (eBioscience) as described by the manufacturer’s instructions. For cultured PBMCs, cells were washed in PBS, blocked in 1:20 TruStain FcX (Biolegend) for 10 min at room temperature, stained with 1:25 Zombie Yellow viability dye (Biolegend) for 15 min at room temperature, and then incubated for 30 min at 4 °C with the following monocyte exhaustion antibody panel: CD11b (AB_2629529, M1/70), CD16 (AB_2744299, 3G8), CD14 (AB_2860953, 63D3), CD200R (AB_2564351, OX-108), CD15 (AB_2927951, W6D3), CD80 (AB_2564407, 2D10), CD86 (Elabscience # E-AB-F1012I, BU63), CX3CR1 (AB_1626276, 2A9-1), HLA-DR (AB_893574, L243), Il-1R2 (AB_2609075, 34141), CD163 (AB_2616879, GHI/61), CD38 (AB_2894562, S17015F), PD-L1 (AB_2871205, MIH1), CD157 (AB_3068112, W21007F), CXCR2 (Novus # NBP1-43338AF532, 5E8-C7-F10), and CCR2 (AB_2800969, K036C2). PBMCs were then washed and resuspended in FACS buffer. Monocyte exhaustion panels were measured using an Aurora 4 L 16 V-14B-10YG-8R (Cytek), while mTOR inhibition, MitoTracker, and Annexin V experiments were measured using a FACSCanto II (BD Biosciences). All flow cytometry data were analyzed in FlowJo (BD Life Sciences).

### Cell proliferation assay

Cell proliferation in DHA-treated BMMCs was measured using a CellTrace™ CFSE Cell Proliferation Kit (Invitrogen) according to the manufacturer’s protocol. Briefly, fresh harvested mouse BMMCs were incubated in a CFSE working solution (1 µL 5 mM CFSE in 3 mL PBS) at room temperature for 8 min. The reaction was diluted 1:1 with FBS to quench the reaction, and cells were washed four times with complete RPMI 1640 medium. Cells were then seeded on 6-well plates at a density of 3 × 10^5^ cells/cm^2^ and cultured as described above. After 5 days, CFSE signal was measured by flow cytometry using a FACSCanto II (BD Biosciences) to evaluate cell proliferation.

### Seahorse assay

Bone marrow cells from 6- to 8-week-old C57BL/6 female mice were seeded at a density of 3 × 10^5^ cells/cm^2^ on a 24-well XF Cell Culture Microplate (Agilent) and cultured for 5 days as described above. Each sample was cultured in technical duplicate or triplicate. Cells were then assayed using an XF Cell Mito Stress Test Kit on a Seahorse XF Flex Analyzer (Agilent) according to the manufacturer’s protocol. To normalize oxygen consumption in each well to total cellular content, following Seahorse run completion each well was lysed with Cytobuster protein extraction reagent (Millipore) and analyzed using a DC protein assay (Bio-Rad) according to the manufacturer’s protocol.

### Cytometric array

Cell culture media from human PBMCs was analyzed using a custom LEGENDplex cytometric array (Biolegend) targeting secreted CCL2, TGF-β1, CCL11, IL-6, IL-10, GM-CSF, and IL-5 according to the manufacturer’s protocol. Samples were analyzed using a FACS Canto II (BD Biosciences).

### Quantitative real-time PCR (qRT-PCR)

Total RNA was extracted using a RNeasy Plus Mini Kit (QIAGEN) and reverse transcribed using a High-Capacity cDNA Reverse Transcription Kit (Applied Biosystems) according to the manufacturer’s protocol. qRT-PCR was performed using Power SYBR Green Master Mix (Thermo Fisher) on a CFX96 Real-Time System C1000 Thermal Cycler (Bio-Rad). Reactions were performed in triplicate, and samples with triplicate variation > 1 C_t_ were excluded from downstream analyses. Relative expression levels were determined using the Pffafl method normalized to the mean of genes *Actb* and *Oaz1*. Melt curve analyses were performed for each sample to exclude false positives. Primer information is available in Supplemental Table 4.

### Bisulfite pyrosequencing

Genomic DNA was prepared from cultured BMMCs using a DNeasy Blood & Tissue Kit (QIAGEN) and bisulfite-treated using an EpiTect Bisulfite Kit (QIAGEN). Regions of interest were PCR amplified using a PyroMark PCR Kit (QIAGEN) and sequenced on a PyroMark Q48 Autoprep instrument. Primer and target region information is available in Supplemental Table 4.

### NAD^+^ assay

An Amplite Fluorimetric cADPR-Ribose Assay Kit (AAT; Bioquest) was used to determine NAD^+^ in cultured BMMCs according to the manufacturer’s protocol. NAD^+^ levels were quantified using a Cytation 3 Cell Imaging Multi-Mode Reader (BioTek).

### Western blot

BMMCs were lysed on 6-well plates in 2% SDS cell lysis buffer containing a cocktail of phosphatase inhibitors 2/3 and protease inhibitor (Sigma). Cell lysates were denatured at 95 °C for 5 min and measured using a DC Protein Assay (Bio-Rad) to determine protein concentrations. Proteins (10–50 µg) were separated on a 10% acrylamide SDS-PAGE gel and wet-transferred onto a PVDF membrane. Membranes were blocked with 5% milk for 1 h at room temperature and then incubated overnight at 4 °C with one of the following primary antibodies: anti-STAT1 (RRID: AB_2198300), anti-phospho-STAT1 (Ser727, AB_2197983), anti-STAT3 (D3Z2G, AB_2629499), anti-phospho-STAT3 (Ser727, AB_331589), anti-p70(S6K) (AB_2269787), anti-PGC1α/β (AB_2809840), anti-phospho-AKT (Ser473, AB_329825), anti-CREB (48H2, AB_331277), anti-phospho-CREB (Ser133, 87G3, AB_2561044), or anti-β-actin (AB_330288). Membranes were then washed in TBST and incubated for 1 h at room temperature in HRP-conjugated anti-rabbit IgG (AB_2099233). Images were developed with ECL detection kit (VWR). Relative protein expressions were quantified with ImageJ software (NIH).

### Statistics

General statistics were performed using Prism version 10.4.2 (Graphpad). Normal distributions were examined using the Shapiro-Wilk test. For normally distributed data, comparisons within control and DHA-treated groups were corrected using Šídák’s method based on the assumption that each comparison is independent of the other. Select samples were analyzed using a Brown-Forsythe and Welch ANOVA with Dunnett’s T3 multiple comparisons test based on observed unequal standard deviation between groups. Modeling of DHA dose-response curves was performed using 4PL nonlinear regressions, and comparisons between DMSO control and 30 µM DHA-treated PBMCs was performed using paired* t* tests. Statistical significance is accepted for adjusted* p*-values < 0.05. No statistical method was used to pre-determine sample size. No data points or animals were excluded from these analyses.

## Results

### DHA inhibits the development of monocyte exhaustion during sepsis

**Fig. 1:**
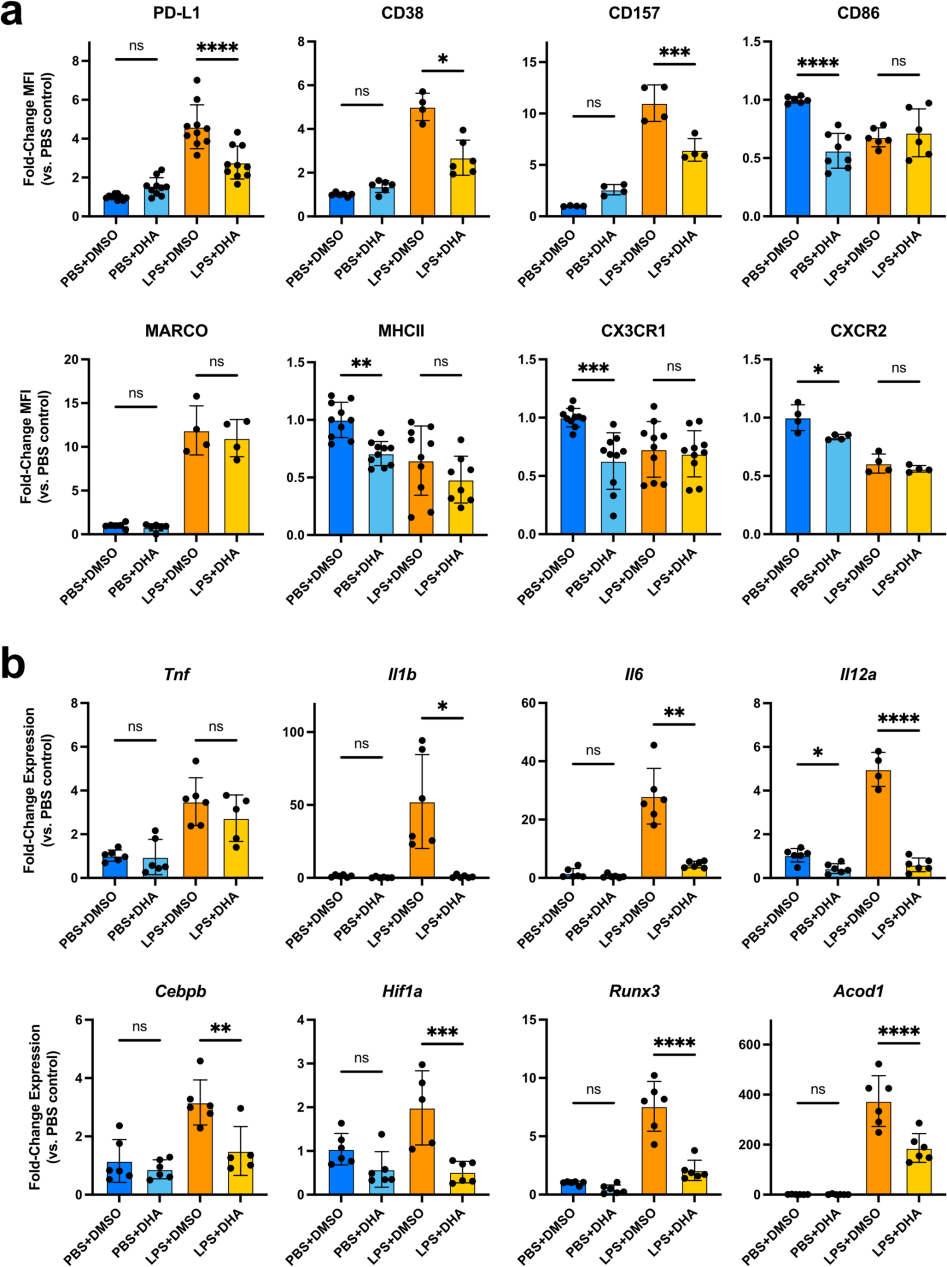
DHA ameliorates monocyte exhaustion and inflammation.** a** Flow cytometry mean fluorescence intensity (MFI) values for monocyte exhaustion markers in cultured BMMCs relative to PBS+DMSO (mean+/−STD; *n* = 4–10; one-way ANOVA with Sidak’s multiple comparisons test; **** p-adj. < 0.0001; *** < 0.001; ** < 0.01; * < 0.05; ns not significant; exact* p* values available in Supplemental Table 2).** b** qRT-PCR for inflammatory cytokines and key transcription factors in cultured BMMCs relative to PBS+DMSO (mean+/−STD; *n* = 4–6; one-way ANOVA with Sidak’s multiple comparisons test, except *Il1b* and *Il6* analyzed by Brown-Forsythe and Welch ANOVA with Dunnett’s T3 multiple comparisons test)

To test the therapeutic potential of DHA intervention on the development of pathogenic innate immune memory during sepsis, we employed an ex vivo sepsis model in which mouse bone marrow monocytic cells (BMMCs) are cultured for 5 days in the presence high-dose lipopolysaccharide (LPS, 100ng/mL) leading to monocyte exhaustion [12]. BMMCs were supplemented for the full culture duration with a physiological 60µM dose of DHA [34]. After 5 days, BMMCs were analyzed by flow cytometry with a 13-factor monocyte exhaustion phenotyping panel. Control DMSO-treated BMMCs challenged with LPS developed the expected CD38^high^;PD-L1^high^;CX3CR1^low^ exhaustion profile (Fig. 1a). By contrast, DHA treatment led to a significant reduction in PD-L1 and CD38 cell surface levels. We also noted a reduction in the levels of CD157, a CD38 paralog thought to regulate intercellular adhesion and transendothelial migration [35]. Interestingly, in PBS control monocytes DHA supplementation reduced CD86, MHCII, CX3CR1, and CXCR2 in a manner analogous to LPS-treated cells, suggesting a general decrease in immune reactivity. This is reflected in a shift from an intermediate monocyte subtype toward the more pro-resolving non-classical subtype (Fig. S2a–b). By contrast, macrophage differentiation and the expression of immune-enhancing molecules MARCO, CD74, and CD200R were unimpacted by DHA treatment (Fig. 1a, S2c–d). We also noted that early DHA intervention was essential for limiting the induction of CD38, CD157, and PD-L1 expression, as delaying DHA treatment until day 4 of BMMC culture had a negligible impact on these markers (Fig. S2e).

Exhausted monocytes are also characterized by persistent secretion of inflammatory cytokines [36]. Whereas *Tnf* expression was unaffected by DHA treatment, the expression of *Il1b*, *Il6*, and *Il12a* was restored to basal levels in LPS-treated BMMCs (Fig. 1b). qRT-PCR for major transcription factors regulating monocyte exhaustion revealed that *Cebpb*, *Hif1a*, and *Runx3* were all significantly downregulated by DHA treatment. Similarly, DHA treatment suppressed the transcription of immunometabolic regulator *Acod1*, which has been recently shown to promote PD-L1 production in septic monocytes [37]. In summary, these results demonstrate that DHA intervention can suppress the development of monocyte exhaustion resulting from septic stress.

### DHA disrupts sepsis-mediated epigenetic reprogramming in monocytes

**Fig. 2:**
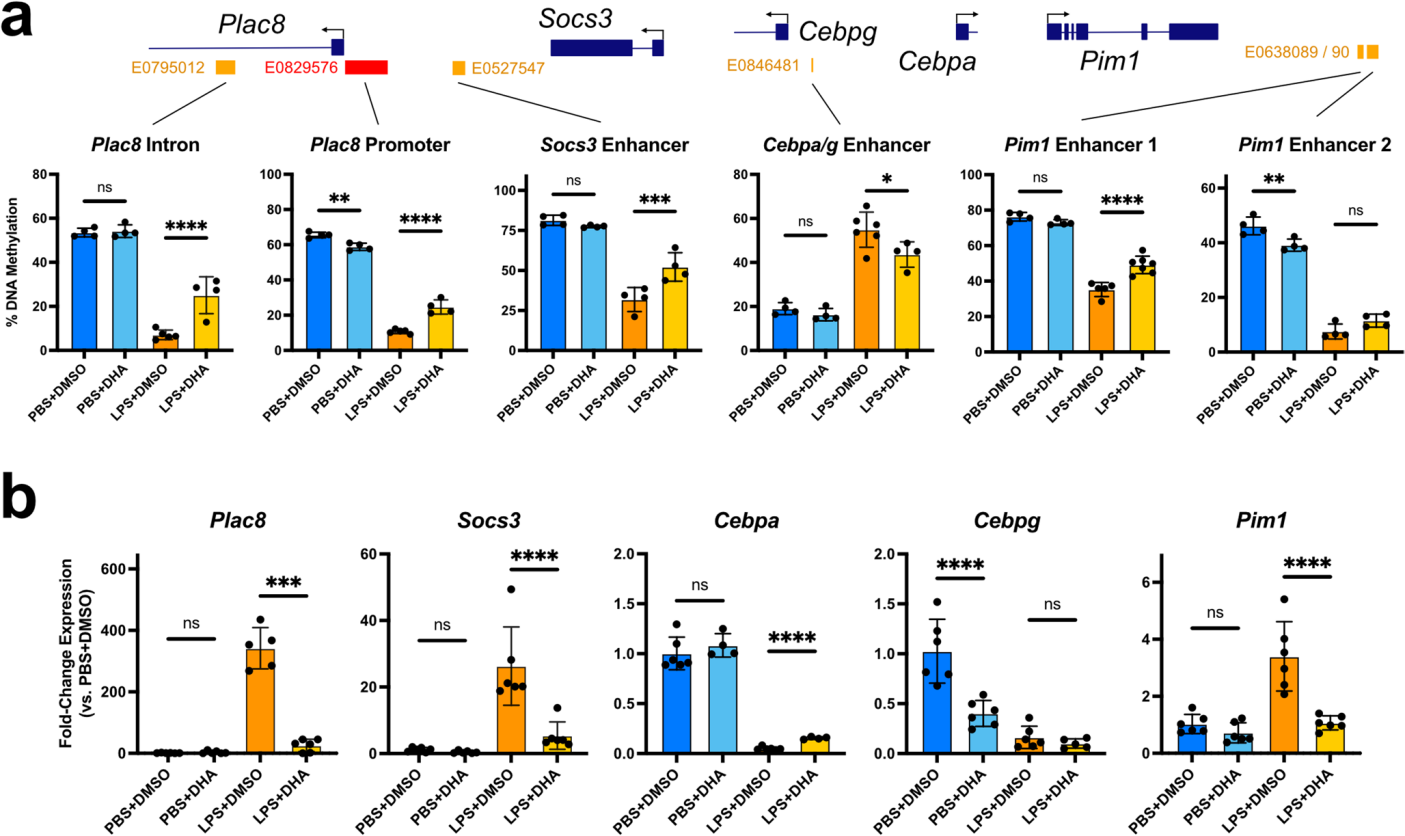
DHA restores epigenetic memory in LPS-exhausted monocytes.** a** Bisulfite pyrosequencing for DNA methylation at major exhaustion DMRs in cultured BMMCs. ENCODE annotated promoters (red) and enhancers (orange) indicated beneath each gene map (mean+/−STD; *n* = 4–6; one-way ANOVA with Sidak’s multiple comparisons test; **** p-adj. < 0.0001; *** < 0.001; ** < 0.01; * < 0.05; ns not significant; exact* p* values available in Supplemental Table 2).** b** qRT-PCR for DMR-linked genes in cultured BMMCs relative to PBS+DMSO (mean+/−STD; *n* = 4–6; one-way ANOVA with Sidak’s multiple comparisons test, except *Cebpa* analyzed by Brown-Forsythe and Welch ANOVA with Dunnett’s T3 multiple comparisons test)

Previous work from our group demonstrated that sepsis promotes DNA methylation reprogramming in exhausted monocytes to reinforce long-term transcriptional memory [11]. We next performed bisulfite pyrosequencing to measure DNA methylation at key differentially methylated regions (DMRs) in LPS-exhausted BMMCs cultured in the presence of DHA. Our results demonstrated a significant increase in DNA methylation at enhancers and promoters for major exhaustion genes *Plac8*, *Socs3*, *Pim1*, and *Ak2* as well as diminished hypermethylation at a shared *Cebpa/g* enhancer following DHA treatment (Fig. 2a, S3a). Altered DNA methylation at these regulatory sites was correlated with significant transcriptional changes in their linked genes (Fig. 2b, S3b). This effect was not observed for DMRs at the *Foxp1* promoter and *Klf4* enhancer, although DHA supplementation did lead to a significant decrease in *Foxp1* expression in both PBS control and LPS-treated BMMCs (Fig. S3a–b). Interestingly, DHA-treatment also increased hypermethylation at an intergenic enhancer linked to *Creb1* and *Klf7* correlating with diminished expression of these two genes. However, transcriptional downregulation was also observed in PBS control cells, suggesting this effect is independent of enhancer DNA methylation. These results demonstrate that DHA treatment can inhibit the formation of epigenetic memory, potentially impacting long-term innate cell behavior.

### Improved mitochondrial respiration and cell survival in DHA-treated septic monocytes mechanistically linked to STAT1/3 Inhibition

**Fig. 3:**
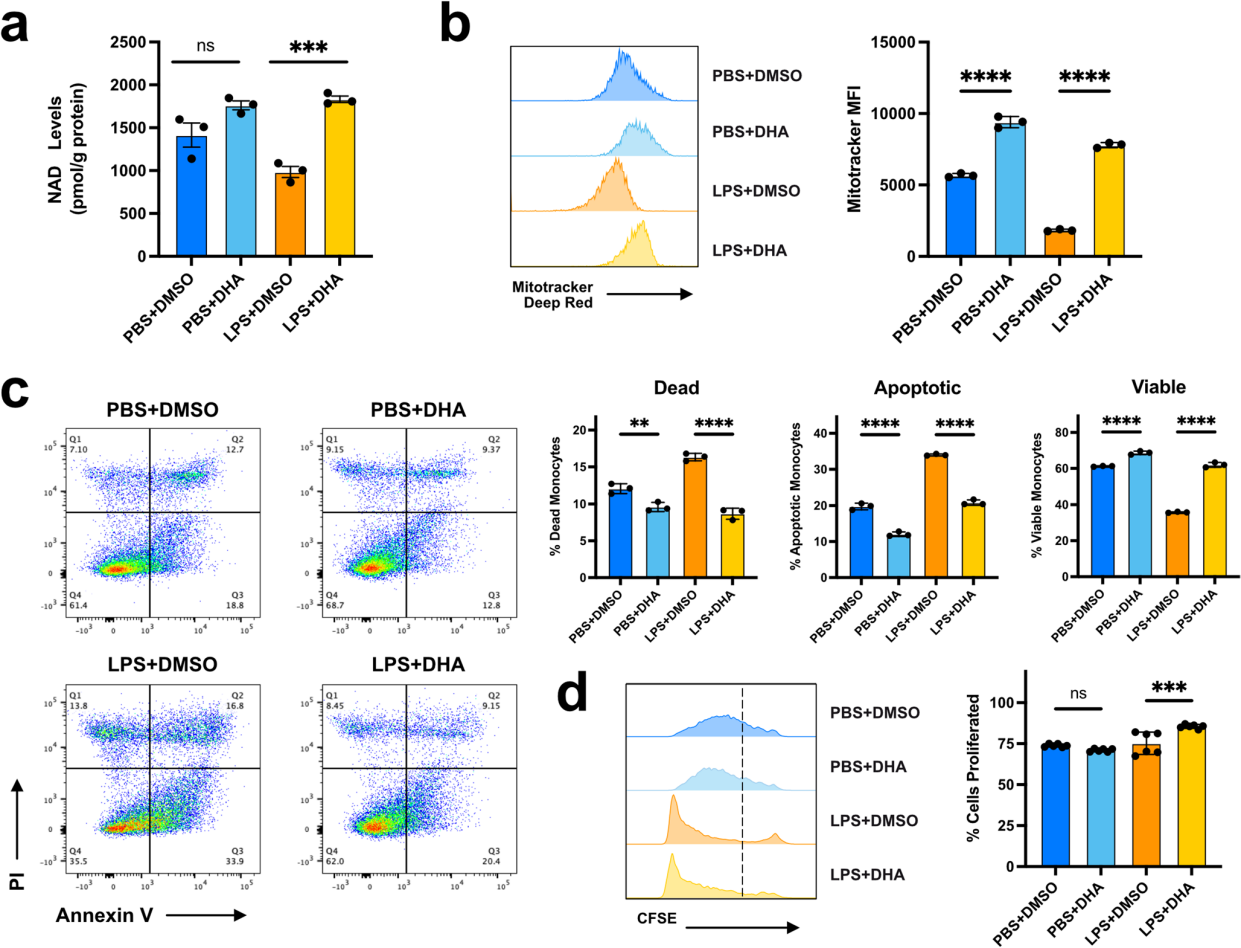
DHA supports mitochondrial respiration and cell survival during LPS exhaustion of monocytes.** a** NAD+ levels in cultured BMMCs normalized to total protein levels (mean+/−STD; *n* = 3; one-way ANOVA with Sidak’s multiple comparisons test; **** p-adj. < 0.0001; *** < 0.001; ** < 0.01; * < 0.05; ns not significant; exact* p* values available in Supplemental Table 2).** b** Flow cytometry for mitochondrial potential staining in cultured BMMCs with Mitotracker (mean+/−STD; *n* = 3; one-way ANOVA with Sidak’s multiple comparisons test).** c** Flow cytometry cell survival in cultured BMMCs. Cells defined as dead (Annexin V^−^ ; PI^+^), apoptotic (Annexin V^+^), or viable (Annexin V^−^ ; PI^−^) (mean+/−STD; *n* = 3; one-way ANOVA with Sidak’s multiple comparisons test).** d** Flow cytometry for cell proliferation in cultured BMMCs. Dotted bar in CFSE histogram plot indicates MFI cutoff for proliferated cells (mean+/−STD; *n* = 3; one-way ANOVA with Sidak’s multiple comparisons test)

Omega-3 supplementation has a variable impact on mitochondrial bioenergetics and cell survival, alternatively promoting reactive oxygen species formation and apoptosis or antioxidant and anti-apoptotic activity in different cellular and disease contexts; such variability may also be attributed to differences in DHA preparation quality, concentration, and bioavailability between studies [38]. Consistent with our previous research, exhausted BMMCs exhibited diminished NAD^+^ levels and mitochondrial membrane potential after 5 days of LPS culture, an effect that was potently reversed by DHA supplementation (Fig. 3a–b) [12, 39]. Analysis of the cells by Seahorse assay revealed that while the basal oxygen consumption rate (OCR) of LPS stimulated cells is greatly compromised in exhausted BMMCs, DHA supplementation restored the OCR to near control levels (Fig. S4a). We next performed Annexin V staining to measure the impact of DHA treatment on cellular viability and observed a significant increase in cell survival in both PBS control and LPS-treated BMMCs (Fig. 3c). We also noted a small but significant increase in cell proliferation in LPS-treated cells (Fig. 3d). Taken together, these results indicate that DHA intervention positively impacts mitochondrial respiration and cell viability during septic stress, further supporting the protective benefit of omega-3 supplementation in limiting monocyte exhaustion.

**Fig. 4:**
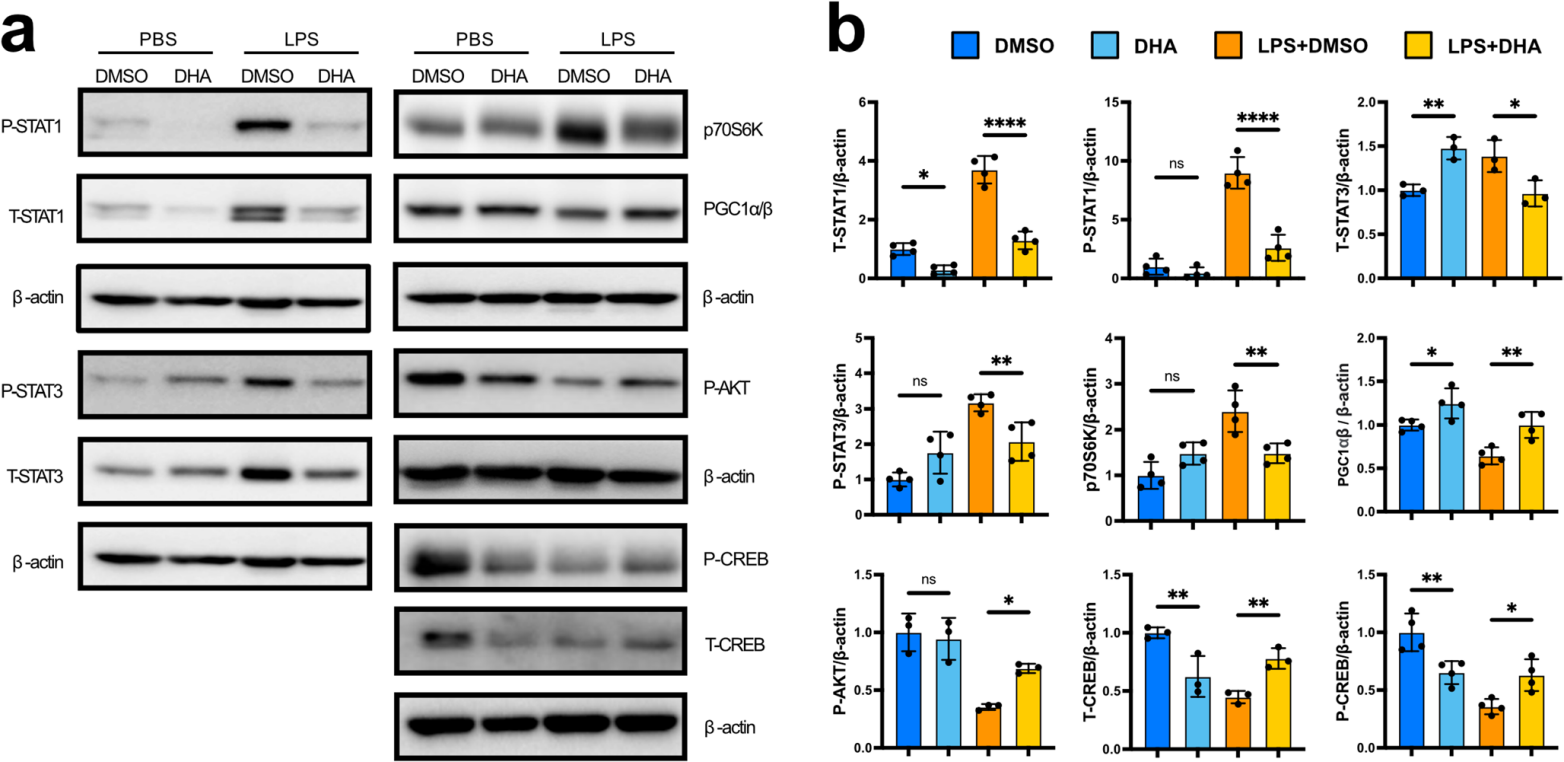
Protective effect of DHA is mechanistically linked to STAT1/3 inhibition in LPS-exhausted monocytes.** a** Representative western blot images for STAT and mTOR signaling factors in PBS control or LPS-challenged BMMCs cultured in the presence of DMSO or 60 µM DHA. Total (T) or phosphorylated (P) protein levels were measured for STAT1, STAT3, AKT, and CREB. Matched β-actin loading controls are presented beneath each set of signaling factors. **b** Quantification of STAT western blot chemiluminescent signal. Relative protein levels were normalized to β-actin. (mean+/-STD; *n* = 3–4; one-way ANOVA with Sidak’s multiple comparisons test; **** p-adj. < 0.0001; *** < 0.001; ** < 0.01; * < 0.05; ns not significant; exact* p* values available in Supplemental Table 2).

Given its therapeutic potential, we next sought to determine the mechanism by which DHA antagonizes exhaustion memory formation in septic monocytes. Previous work from our group demonstrated that STAT1 activation downstream of TICAM2 signaling is a major driver of the exhaustion phenotype [12]. Consistent with this model, DHA intervention substantially reduced both total and phosphorylated STAT1 levels in LPS-treated BMMCs, with a similar effect observed for STAT3 (Fig. 4a–b). DHA treatment also increased total and phosphorylated CREB levels in LPS-treated BMMCs, thereby supporting a major signaling axis mediating the pro-resolving effect of SPMs [40, 41]. Based on previous literature reporting altered AKT-mTOR signaling in DHA treated cells, we also measured the levels of AKT-mTOR signaling components and noted that DHA treatment simultaneously increased AKT phosphorylation in LPS-treated BMMCs while decreasing the total levels of downstream mTOR component p70S6K (Fig. 4a–b) [27, 42]. Finally, we found that DHA treatment restore PGC1α/β levels in LPS-treated cells, potentially accounting for the observed improvement in mitochondrial function [43]. 

Increased AKT-mTOR signaling in DHA-treated BMMCs was unexpected given our recent study demonstrating that mTOR promotes monocyte exhaustion during sepsis [33]. To test the influence of this elevated mTOR signaling on exhaustion phenotype development, we cultured DHA-treated BMMCs in the presence of mTOR inhibitor AZD2014. Consistent with our recent study, mTOR inhibition led to a combinatorial improvement in CD38 and PD-L1 suppression and CD86 activation in DHA-treated cells challenged with LPS (Fig. S4b). Similar effects were also observed for cell viability and proliferation (Fig. S4c–d). Thus, elevated mTOR signaling in DHA-treated septic monocytes interferes with the protective effect of DHA on monocyte exhaustion, suggesting the therapeutic potential of DHA can be augmented when combined with mTOR inhibition.

Cumulatively, these results support a protective role for DHA treatment on monocyte survival and metabolic function during sepsis. This effect is independent of DHA-mediated activation of mTOR signaling, instead proceeding through a convergence of STAT1/3 inhibition, CREB activation, and elevated PGC1α/β levels.

### DHA treatment alters immunoreactivity and chemotactic potential in septic patient monocytes

**Fig. 5:**
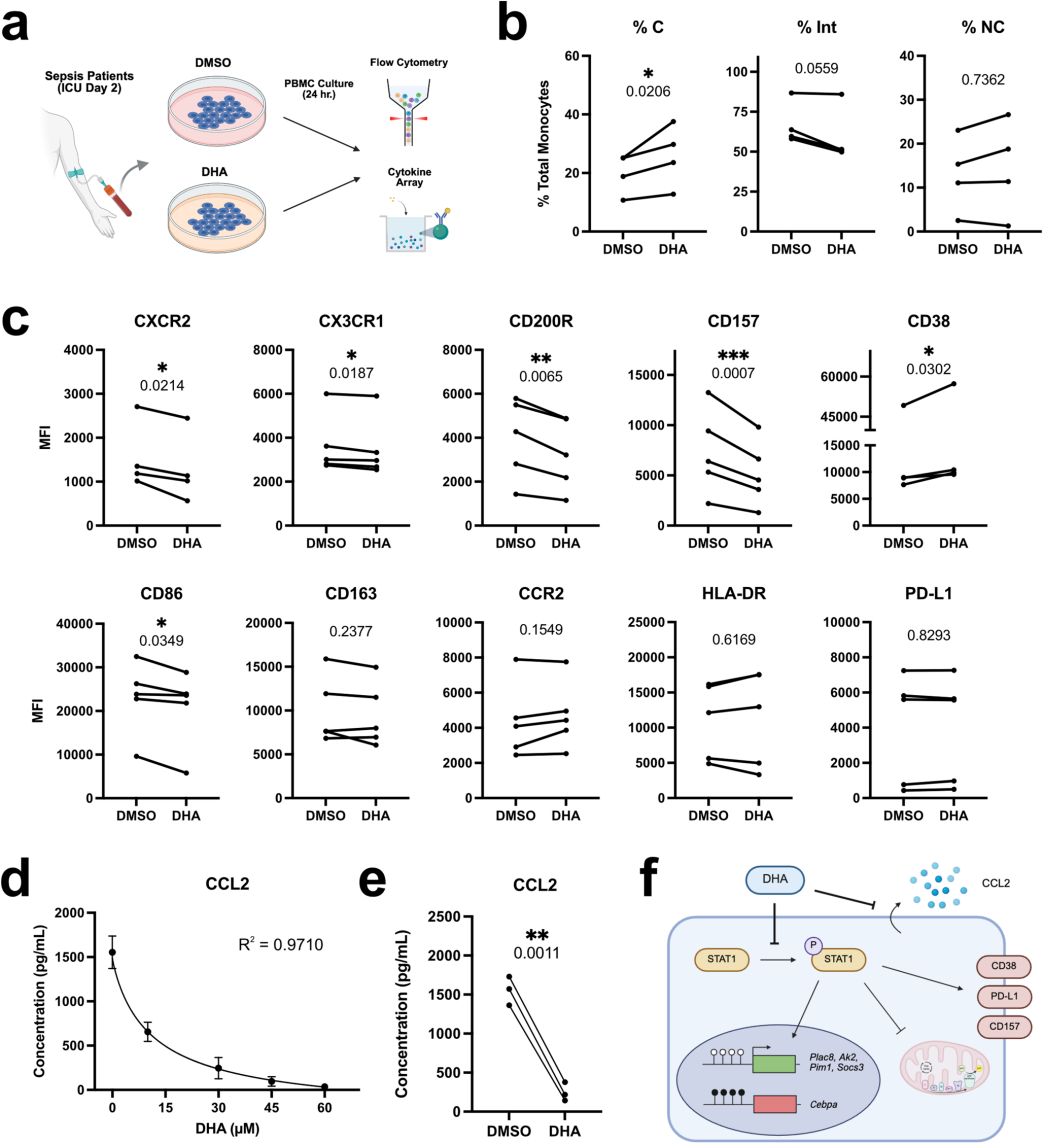
DHA treatment dampens inflammatory stress and immune activation in septic patient monocytes.** a** Experiment outline for DHA treatment of septic patient peripheral blood monocytes.** b** Flow cytometry analysis of monocyte subtypes in cultured PBMCs treated with DMSO or 30µM DHA (*n* = 4; paired t test; **** p-adj. < 0.0001; *** < 0.001; ** < 0.01; * < 0.05).** c** Flow cytometry for immune signaling markers in cultured PBMCs (*n* = 4–5; paired* t* test).** d** Dose-response curve for secreted CCL2 in cultured PBMC media for varying concentrations of DHA treatment (mean+/-STD indicated for each DHA dose; *n* = 3; 4PL nonlinear regression).** e** Secreted CCL2 levels in cultured PBMCs treated with DMSO or 30µM DHA (*n* = 3; paired* t* test).** f** Model for DHA-mediated inhibition of monocyte exhaustion

Our experiments in mouse septic BMMCs suggest that DHA treatment has the potential to inhibit the formation of pathogenic innate immune memory states resulting from septic stress. To establish the translatability of these findings, we tested the effect of short-term DHA supplementation on human peripheral blood mononuclear cell (PBMC) cultures derived from sepsis patients (Table S1). PBMCs collected from septic patients two days after ICU admission were treated for 24 h with variable concentrations of DHA and analyzed by flow cytometry with a 16-factor monocyte exhaustion phenotyping panel (Fig. 5a). Strikingly, we observed dose-dependent alterations in monocyte CD38, CD157, CXCR2, CD200R, CD86, and CX3CR1 levels in response to DHA supplementation (Fig. S5a–b). These changes were correlated with a shift in monocytes from the CD14^+^;CD16^+^ intermediate subtype toward the CD14^+^;CD16^−^ classical subtype (Fig. 5b). Focusing on a minimum effective dose of 30µM DHA, CXCR2, CX3CR1, CD157, and CD86 levels were all significantly reduced in a manner analogous to mouse BMMC culture (Fig. 5c). Furthermore, although monocyte PD-L1 levels were unimpacted by short-term DHA supplementation, we did note a significant reduction in immunosuppressive CD200R expression following DHA treatment [44]. By contrast, monocyte CD38 levels slightly increased following DHA treatment, suggesting that like mouse BMMCs, early and sustained DHA intervention is essential to mitigate CD38 upregulation during sepsis.

Given the rapidly shifting immune landscape of sepsis patients, we next tested the effect of DHA treatment on PBMCs isolated through serial blood draws from a patient 2, 4, and 6 days after ICU admission (Fig. S5c). Whereas the impact of DHA supplementation on CD200R, CX3CR1, and CD157 was consistent across all tested time points, the observed reduction in CD86 was lost by day 6, and the effect on CXCR2 was reversed by day 4. In addition, CD38 upregulation gradually worsened with sepsis progression, further emphasizing the necessity of early DHA intervention in limiting monocyte dysregulation.

To determine the effect of DHA supplementation on cytokine signaling by septic monocytes, we next used a 7-factor cytometric array to measure secreted cytokine levels in PBMC cultures following 24 h of DHA treatment. Whereas DHA treatment was not shown to impact the levels of TGF-β1, CCL11, IL-6, IL-10, GM-CSF, or IL-5 in suspension, we noted a profound dose-dependent disruption in chemoattractant ligand CCL2 secretion in DHA-treated cultures (Fig. 5d–e, Table S3). This effect was especially pronounced on PBMCs collected 4 and 6 days after ICU admission, which secreted at least seven-fold more CCL2 at baseline (Fig. S5d).

Taken together, these results support the translatability of our mouse model to human patients, thus highlighting the potential of DHA supplementation in limiting chronic monocyte dysregulation and immune memory after septic injury (Fig. 5f).

## Discussion

In our current study, we demonstrate that DHA supplementation can interfere with the establishment of monocyte exhaustion, thereby limiting acute and chronic immune dysregulation following sepsis. This effect is exerted both at the level of transcription, as observed in the downregulation of inflammatory cytokines and transcription factors such as *Hif1a* and *Runx3*, and at the level of epigenetics, as observed in altered DNA methylation at gene regulatory elements linked to exhaustion memory. Further improvements in monocyte cellular health are also notable, including restored mitochondrial membrane potentials and increased cell viability. The net effect of these alterations is mitigated monocyte immunoreactivity with the potential to limit pathogenic inflammation and immunoparalysis during sepsis recovery.

Much like T cells, monocytes can develop a form of immune exhaustion characterized by paradoxical pro-inflammatory and immunosuppressive activity, reduced immune effector function, and impaired differentiation into macrophages and dendritic cells [12, 13]. These cells are distinguished by high cell surface levels of two factors: CD38, a type II transmembrane glycoprotein with roles in NAD metabolism and immune inflammation, and PD-L1, an immunosuppressive ligand that drives T cell exhaustion and immunoparalysis after sepsis [35, 45]. Our results demonstrate that early DHA intervention blocks the induction of these two molecules, thereby stabilizing cellular NAD^+^ pools and limiting immunosuppressive behavior. Given that inhibition of either CD38 or PD-L1 has shown therapeutic potential in experimental sepsis models, DHA represents a safe and effective means of targeting both molecules to limit long-term negative impact of monocyte exhaustion on sepsis recovery [33, 46, 47]. 

Previous work from our group demonstrated that TICAM2-mediated activation of STAT1 signaling leads to monocyte exhaustion during sepsis [10, 12]. Consistent with reports that DHA suppresses STAT1 signaling in LPS-stimulated macrophages, our current study found that DHA treatment potently suppresses STAT1 activation, thereby interfering with the establishment of monocyte exhaustion during sepsis [20]. Interestingly, DHA also suppresses STAT3 activation in cultured BMMCs, as has been reported in dendritic cells and numerous cancer cell lines [48–51]. Whereas STAT1 and STAT3 modulate innate cell behavior through functionally opposed mechanisms of immune activation and immunosuppression, their convergent activity in exhausted monocytes likely underlies their paradoxical behavior in a manner similar to that observed in myeloid derived suppressor cells (MDSCs) [52, 53]. Critically, both STAT pathways have been implicated in sepsis pathogenesis, underscoring the therapeutic potential for DHA supplementation in limiting septic injury [54, 55]. 

While our results support a protective role for DHA-mediated STAT1 inhibition in the suppression of monocyte exhaustion, the mechanism by which it achieves this remains to be determined. In its unmetabolized form, DHA readily incorporates into the cell membrane, potentially impacting the organization of immune signaling receptors. Although previous research demonstrated that DHA treatment has a negligible effect on macrophage TLR4 and CD14 membrane organization, a separate study found that DHA interferes with IFNγ receptor colocalization with lipid rafts, resulting in impaired STAT1 phosphorylation in macrophages upon *M. tuberculosis* infection [56, 57]. Alternatively, oxidized DHA derivatives have been shown to activate PPARα/γ to suppress NF-κB signaling, potentially disrupting crosstalk between the STAT1 and NF-κB regulating pro-inflammatory transcription [58–61]. Finally, conversion of DHA into pro-resolving lipid species by monocytic lineage cells may interfere with inflammatory signaling upstream of STAT1 activation. Monocytic lineage cells can convert DHA into maresin-class SPMs (e.g. MaR1), supporting pro-resolving immune activity through suppressed STAT1 and STAT3 inflammatory signaling [62]. Additionally, hydroxy docosohexaenoic acids (HDHA), intermediate metabolites in the conversion of DHA to D-series resolvins and maresins, exhibit their own pro-resolving activity, thus necessitating further metabolipidomic approaches to mechanistically link SPM biosynthesis to the observed effects on monocyte exhaustion inhibition [24, 63–65]. 

In contrast to adaptive immune memory, innate immune memory relies on reprogramming of the cellular epigenetic landscape to alter responses to future immune stimulation [13, 15]. This epigenetic memory persists upwards of a year after the initial inflammatory event and is likely a major contributor to post-sepsis PICS [13, 65]. Whereas most work on innate immune memory focused on the contribution of histone modifications to transcriptional memory, recent work from our own group and others have implicated DNA methylation as an important contributor to innate memory following sepsis [11, 66]. In the case of monocyte exhaustion, we reported significant DNA hypomethylation at gene regulatory elements promoting the expression of *Plac8*, *Socs3*, and *Pim1* that is reversed by DHA treatment of septic BMMCs. *PLAC8* is notable as the most strongly upregulated gene in peripheral blood monocytes of sepsis and severe Covid-19 patients; while its role in monocytes is unclear, PLAC8 has been implicated as a mediator of phagocytosis, pro-inflammatory cytokine signaling, and cell survival and proliferation [67–70]. SOCS3, by contrast, is a well-characterized suppressor of inflammatory cytokine signaling that operates in a STAT- or cytokine-induced negative feedback loop [71]. PIM1 is a proto-oncogene serine/threonine kinase with roles in myeloid pro-inflammatory NF-κB signaling and metabolism [72, 73]. Notably, PIM1 promotes macrophage chemotaxis and M2 polarization in non-small cell lung cancer via CCL2 transactivation, potentially linking DHA-mediated *Pim1* suppression to diminished CCL2 secretion observed in septic patient PBMCs [74]. 

In our previous work, we demonstrated that immunophenotypic alterations observed in our mouse model of monocyte exhaustion are recapitulated in sepsis patients, with CD38^high^; CX3CR1^low^; MHCII^low^ monocytes predominating among PBMCs within 5 days of ICU admission [9]. To further assess the translatability of our findings, we tested the impact of short-term DHA supplementation on septic patient PBMCs and observed a strong correlation with our rodent model. Of particular note, DHA treatment in both models resulted in a substantial decrease in CD157 cell surface levels; while the role of CD157 in sepsis is largely unexplored, previous work demonstrated that CD157 is a major regulator of leukocyte transendothelial migration, and thus CD157 inhibition would be expected to limit septic monocyte infiltration into peripheral organs and tissues [75–77]. Compounding this effect, DHA treatment fully ablated CCL2 secretion by cultured PBMCs; given that leukocyte recruitment via CCL2 is a major mechanism of sepsis-driven organ injury, this effect would further contribute to the protective benefit of DHA supplementation during sepsis treatment [78, 79]. 

One notable point of departure between our two model systems was the impact of DHA treatment on monocyte CD38 levels, with a dose-dependent increase in CD38 observed in septic PBMCs. This incongruity likely stems from two sources. First, short-term DHA treatment was ineffective in curbing CD38 upregulation in mouse BMMCs, and thus longer DHA intervention may be necessary for successful CD38 inhibition in human monocytes. Second, serial blood draws from a sepsis patient indicates that the effect of DHA on CD38 upregulation worsens as sepsis progresses, again reaffirming the need for early intervention when targeting monocyte exhaustion. Given the known role of CD38 as a major inflammatory mediator in septic monocytes, these are important considerations when constructing DHA treatment regimens for future studies [33, 47]. 

Several limitations are notable in our study. First, alterations in DNA methylation regulating long-term exhaustion memory are observed at thousands of sites across the genome in septic monocytes. Although our study assayed differential methylation at only select genes involved in sepsis pathogenesis, we anticipate many more regions were impacted than explored here. Likewise, while this study focused on DNA methylation as an epigenetic driver of long-term septic memory, alterations in chromatin remodeling and histone modification are likely also important mediators of the protective effects of DHA on monocyte exhaustion. Finally, because we were unable to test the effect of direct DHA supplementation on septic patients, our experiments with cultured PBMCs assess only the impact of post hoc DHA exposure in resolving features of monocyte exhaustion. These experiments were intended to test the translatability of our rodent model rather than the impact of DHA intervention on chronic monocyte exhaustion memory in septic patients, which will require further study.

Omega-3 supplementation has shown tremendous potential in limiting sepsis risk in pre-morbid and hospitalized adults [80, 81]. Although omega-3 treatment of sepsis patients has not been shown to impact mortality, its effect on post-sepsis recovery remains largely untested [29, 30]. Our study demonstrates that DHA supplementation blocks the establishment of monocyte exhaustion memory, potentially altering the long-term behavior of septic monocytes. These results argue for a reevaluation of omega-3 intervention not for the treatment of acute septic injury, but rather for the prevention of chronic immune dysregulation in sepsis survivors.

## Supplementary Information

Below is the link to the electronic supplementary material.

Supplementary Material 1

Supplementary Material 2

Supplementary Material 3

Supplementary Material 4

Supplementary Material 5

Supplementary Material 6

## Data Availability

Data will be made available on request.
