# Supplementary material for: Docosahexaenoic acid supplementation inhibits monocyte exhaustion memory formation during sepsis
Source: Inflamm Res. 2026 Feb 18;75(1):40. doi: 10.1007/s00011-026-02194-w (PMC12913356; doi:10.1007/s00011-026-02194-w)
Supplement: Supplementary file 1 — Supplementary Material 1 [file 11_2026_2194_MOESM1_ESM.pdf]

Supplemental Information

Docosahexaenoic acid supplementation inhibits monocyte exhaustion memory formation during sepsis

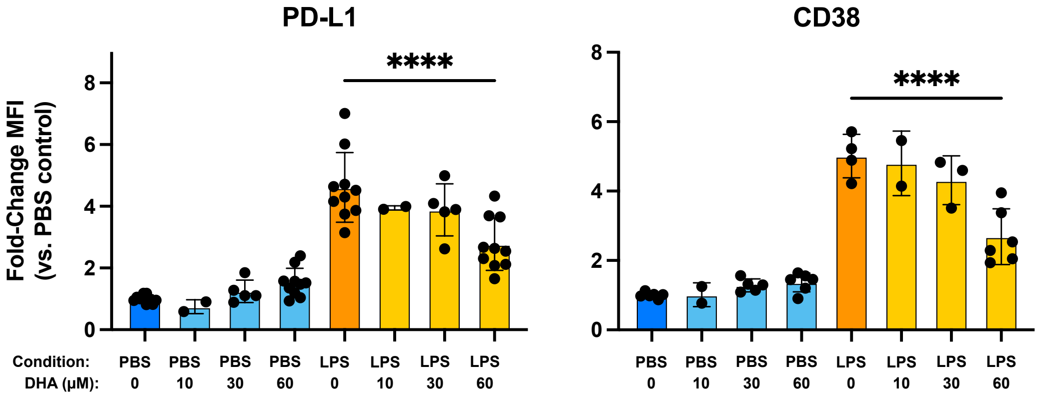

Supplemental Figure 1 – DHA dose determination for mouse BMMC culture. Flow cytometry analysis for exhaustion markers in cultured BMMCs relative to PBS+DMSO. 60µM DHA was used for all subsequent experiments (mean+/-STD; n=2-10; one-way ANOVA with Sidak's multiple comparisons test; **** p-adj. < 0.0001; differences between groups not significant unless specified; exact p values available in Supplemental Table 2).

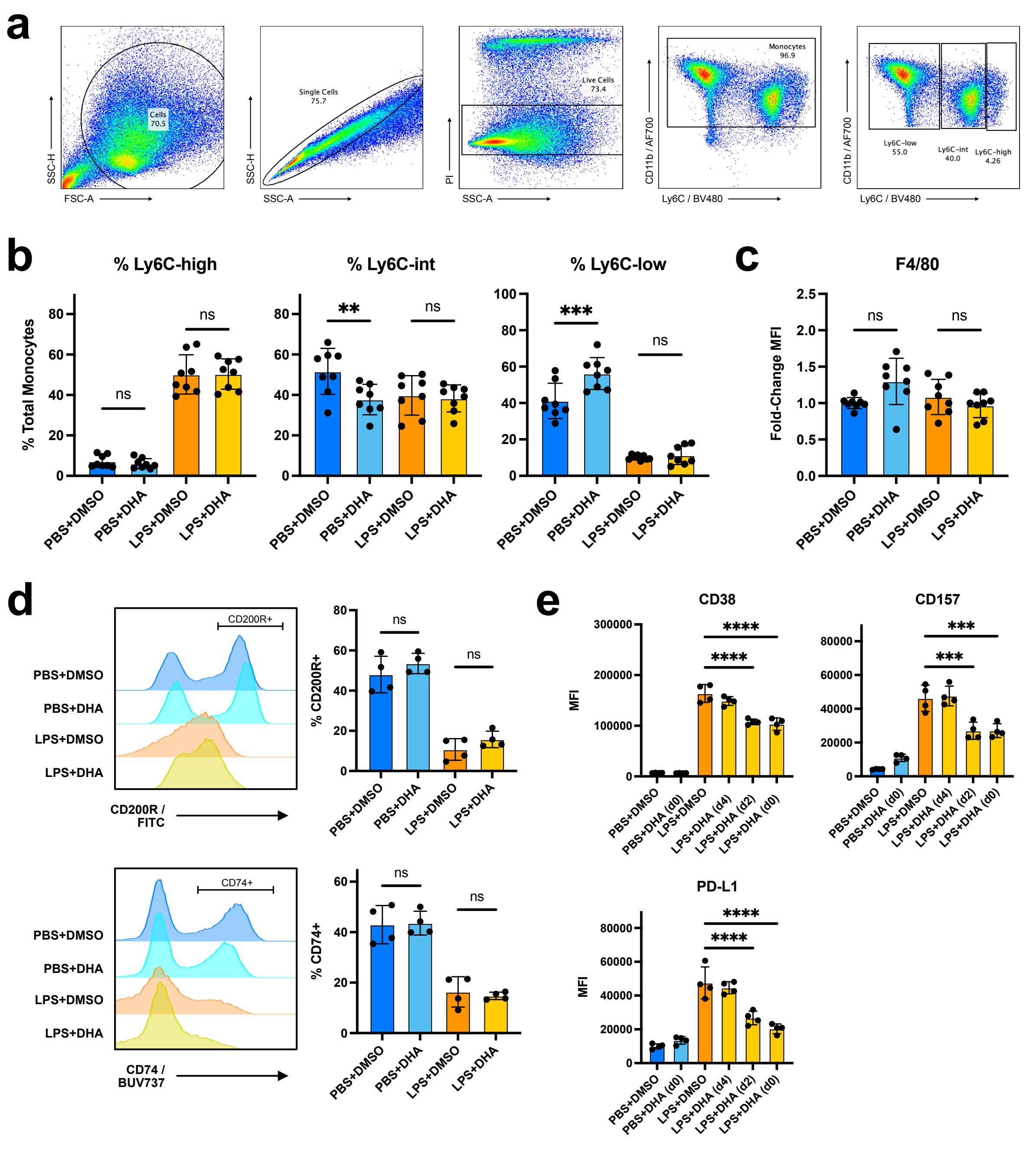

Supplemental Figure 2 – Extended analysis of monocyte exhaustion in DHA-treated BMMCs. (a) Flow cytometry gating strategy for monocyte subtypes in cultured BMMCs. **(b)** Monocyte subtypes in cultured BMMCs (mean+/-STD; n=8; one-way ANOVA with Sidak's multiple comparisons test; **** p-adj. < 0.0001; *** < 0.001; ** < 0.01; * < 0.05; ns not significant; exact p values available in Supplemental Table 2). **(c)**

Flow cytometry analysis of macrophage marker F4/80 in cultured BMMCs relative to PBS+DMSO (mean+/-STD; n=8; one-way ANOVA with Sidak's multiple comparisons test). **(d)** Flow cytometry analysis of $CD74^+$ and $CD200R^+$ cells in cultured BMMCs (mean+/-STD; n=4; one-way ANOVA with Sidak's multiple comparisons test). **(e)** Flow cytometry analysis of monocyte exhaustion markers in cultured BMMCs treated with DHA for varying lengths of time. Cells under continuous PBS control or LPS stimulation were supplemented with 60μM DHA starting on day 0 (d0), day 2 (d2), or day 4 (d4) of BMMC culture (mean+/-STD; n=4; one-way ANOVA with Sidak's multiple comparisons test; differences between groups not significant unless specified).

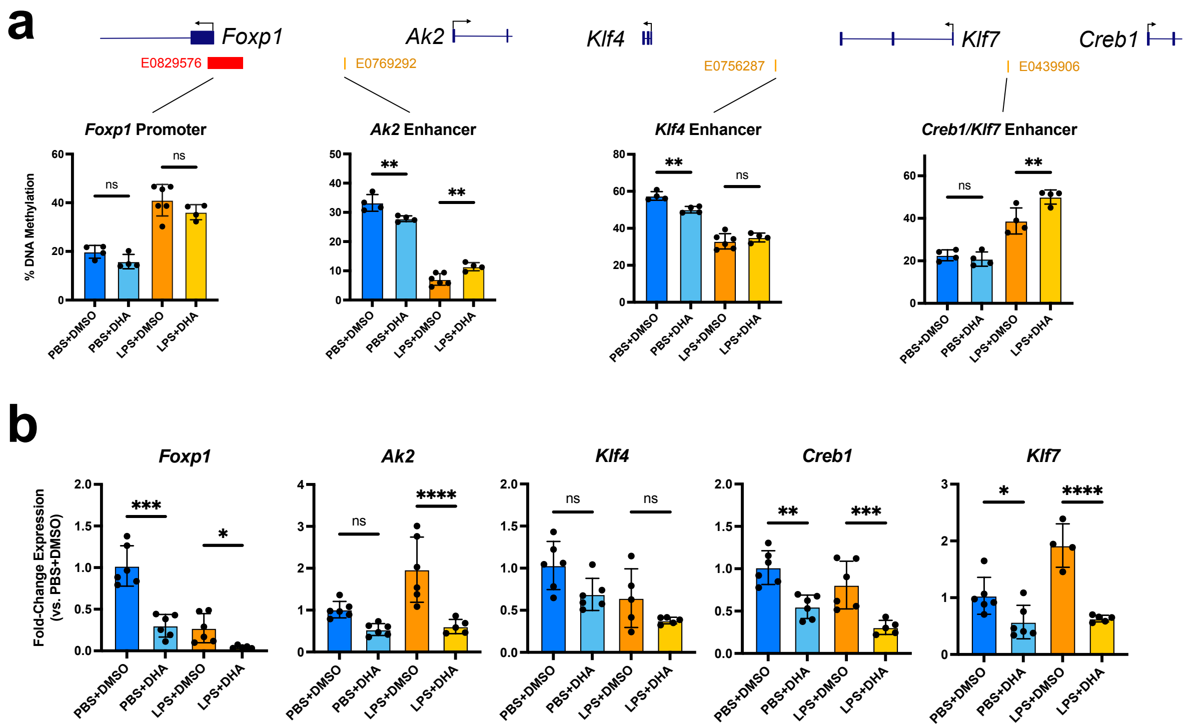

Supplemental Figure 3 – Extended analysis of DNA methylation in DHA-treated BMMCs. (a) Bisulfite pyrosequencing for DNA methylation at major exhaustion DMRs in cultured BMMCs. ENCODE annotated promoters (red) and enhancers (orange) indicated beneath each gene map (mean+/-STD; n=4-6; one-way ANOVA with Sidak's multiple comparisons test; **** p-adj. < 0.0001; *** < 0.001; ** < 0.01; * < 0.05; ns not significant; exact p values available in Supplemental Table 2). **(b)** qRT-PCR for DMR-linked genes in cultured BMMCs relative to PBS+DMSO (mean+/-STD; n=4-6; one-way ANOVA with Sidak's multiple comparisons test, except *Foxp1* and *Klf4* analyzed by Brown-Forsythe and Welch ANOVA with Dunnett's T3 multiple comparisons test).

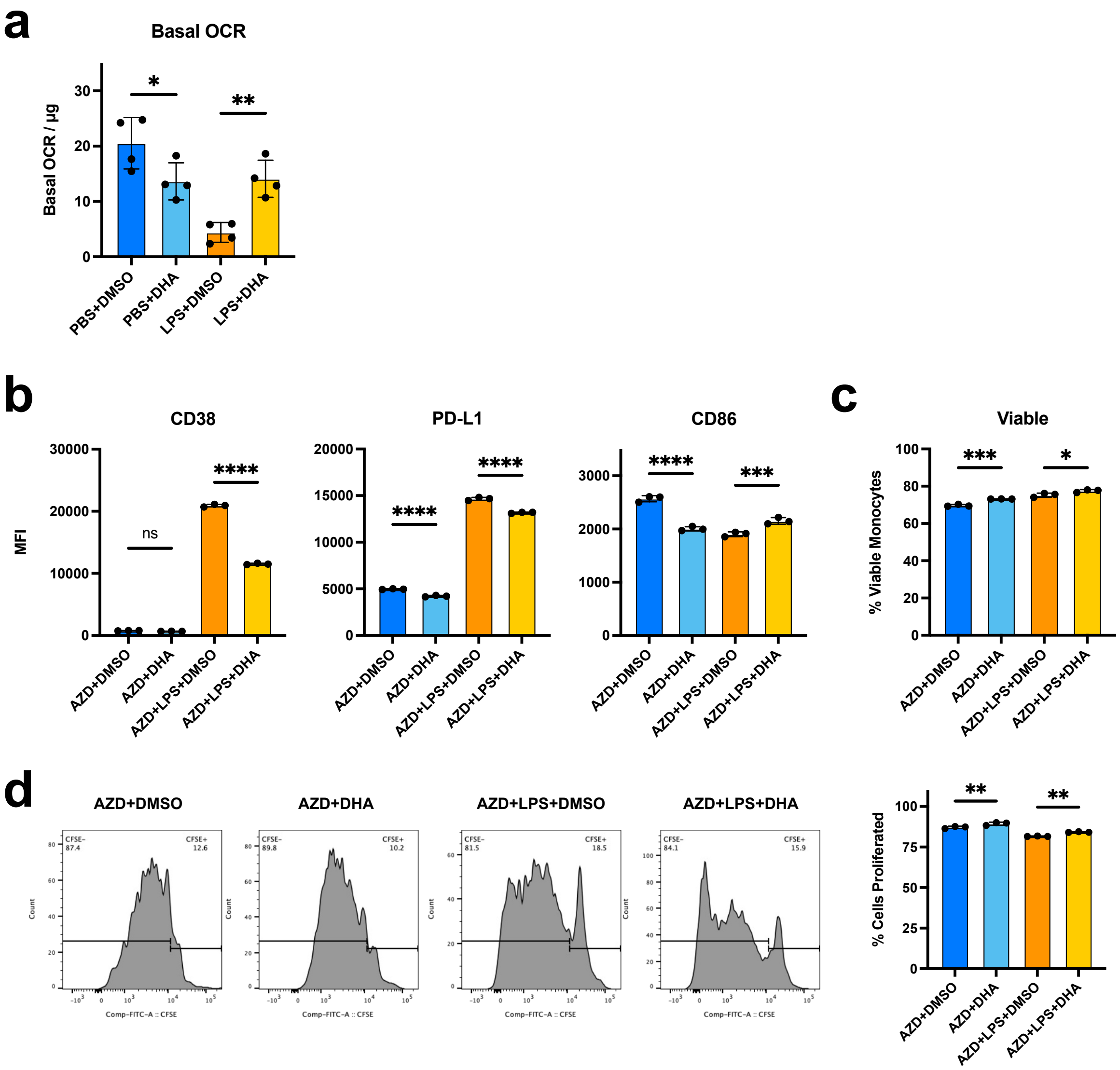

Supplemental Figure 4 – Extended analysis of mitochondrial respiration and mTOR signaling in DHA-treated BMMCs. (a) Seahorse assay for the basal oxygen consumption rate (OCR) in cultured BMMCs normalized to total cellular content. Individual data points represent the average of 2-3 technical replicates (mean+/-STD; n=4; one-way ANOVA with Sidak's multiple comparisons test; **** p-adj. < 0.0001; *** < 0.001; ** < 0.01; * < 0.05; ns not significant; exact p values available in Supplemental Table 2). **(b)** Flow cytometry mean fluorescence intensity (MFI) values for monocyte exhaustion markers in cultured BMMCs treated with mTOR inhibitor AZD2014 (AZD)

and DHA (mean+/-STD; n=3; one-way ANOVA with Sidak's multiple comparisons test).

(c) Flow cytometry cell survival in AZD-treated BMMCs. Viable cells defined as Annexin V^- ; PI^- (mean+/-STD; n=3; one-way ANOVA with Sidak's multiple comparisons test).

(d) Flow cytometry for cell proliferation in AZD-treated BMMCs. MFI cutoff for proliferated cells indicated (mean+/-STD; n=3; one-way ANOVA with Sidak's multiple comparisons test).

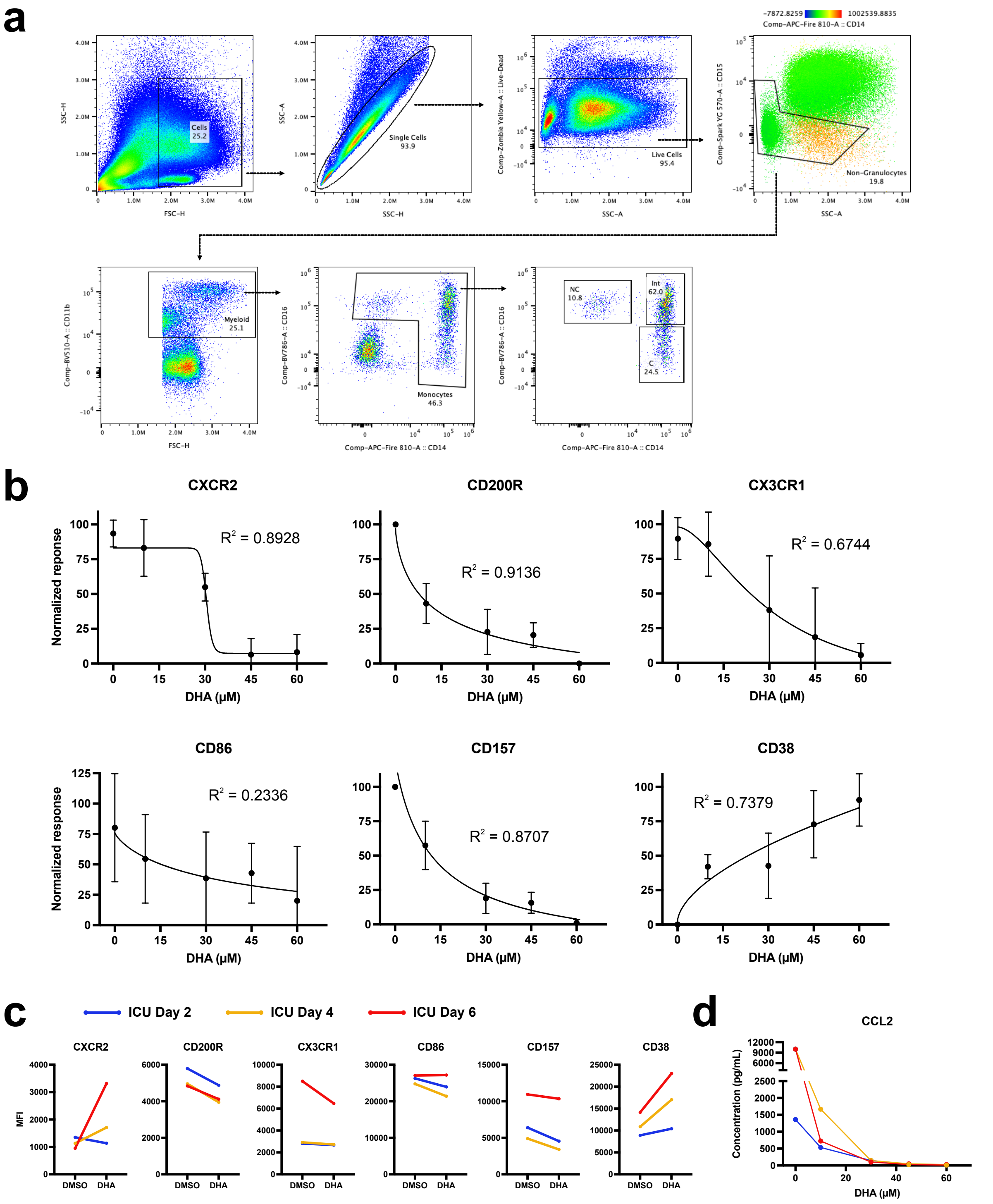

Supplemental Figure 5 – Extended analysis of DHA treatment of sepsis patient PBMCs. (a) Flow cytometry gating strategy for monocyte subtypes in cultured PBMCs from sepsis patients. **(b)** Flow cytometry dose-response curves for immune signaling

markers in cultured PBMCs for varying concentrations of DHA treatment. Normalized responses were calculated based on the minimum and maximum MFI values observed in each patient for a given marker (mean+/-STD indicated for each DHA dose; n=4-5; 4PL nonlinear regression). **(c)** Time course analysis of DHA treatment of cultured PBMCs from a septic patient collected 2, 4, or 6 days after ICU admission. **(d)** Dose-response curve of secreted CCL2 levels in cultured PBMCs from a septic patient collected 2, 4, or 6 days after ICU admission.
