# Supplementary material for: Docosahexaenoic acid supplementation inhibits monocyte exhaustion memory formation during sepsis
Source: Inflamm Res. 2026 Feb 18;75(1):40. doi: 10.1007/s00011-026-02194-w (PMC12913356; doi:10.1007/s00011-026-02194-w)
Supplement: Supplementary file 6 — Supplementary Material 6 [file 11_2026_2194_MOESM6_ESM.pdf]

Uncropped western blot

The bands marked with red rectangles correspond to the western blot analyses presented in this study. For each uncropped blot, the associated main or supplementary figure is indicated. Representative results from three or four independent biological replicates are shown.

Related to Figure 4

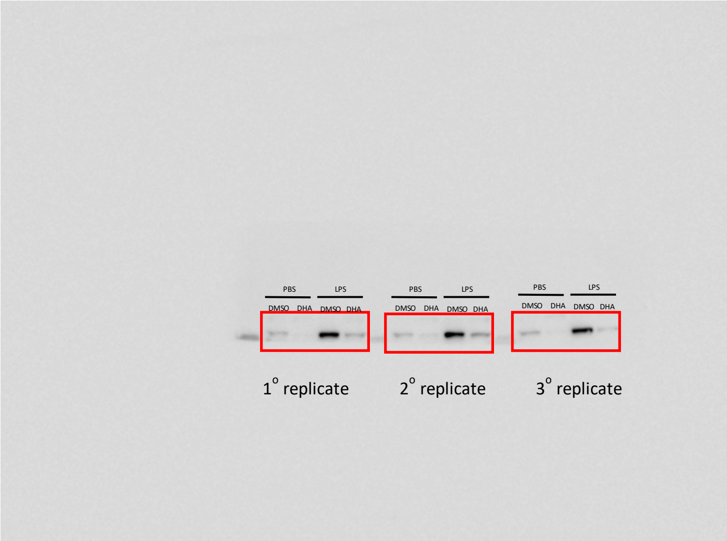

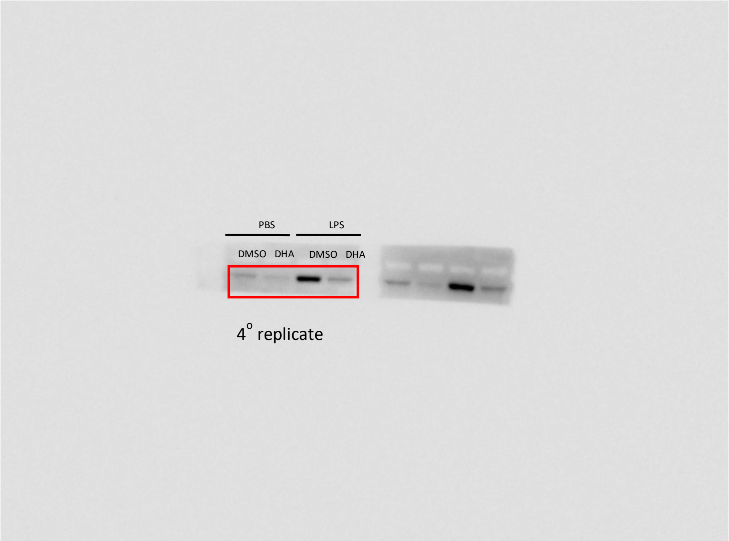

P-STAT1 MW 84kDa

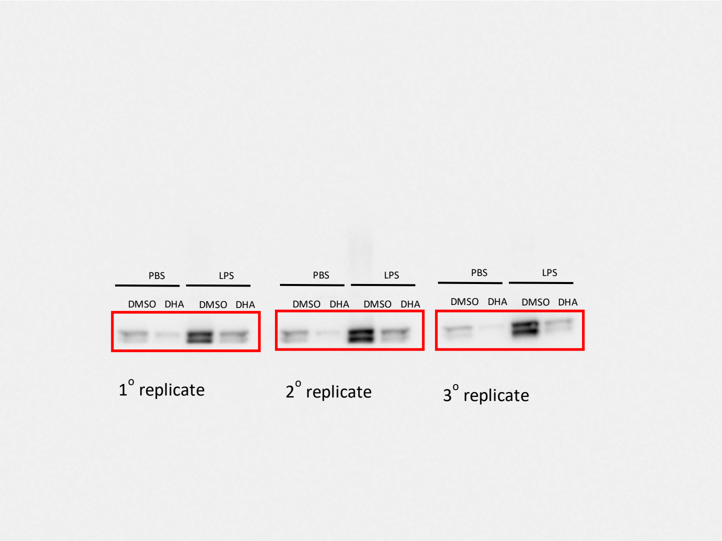

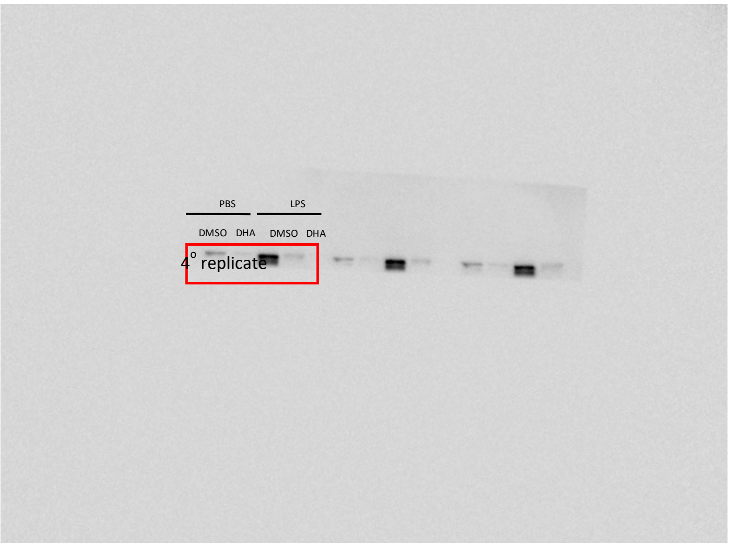

T-STAT1 MW 84, 91kDa

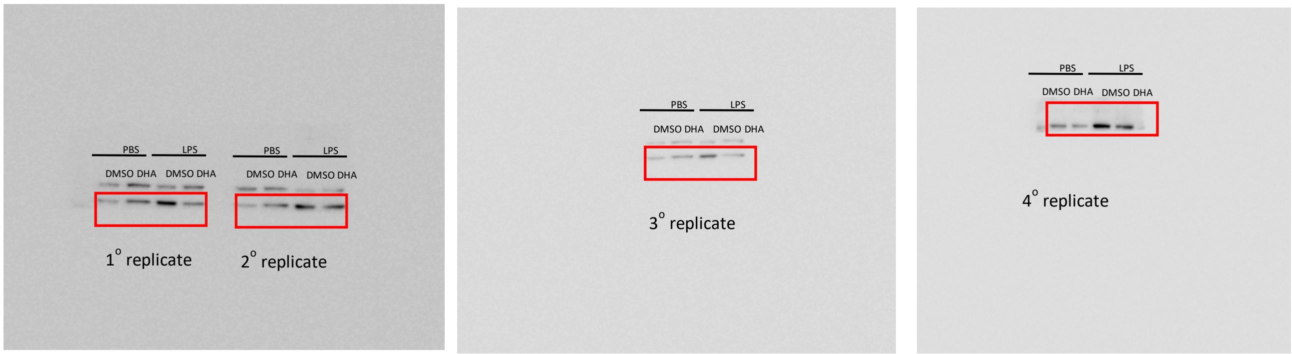

(Membrane was cut from middle)

P-STAT3 MW 79kDa

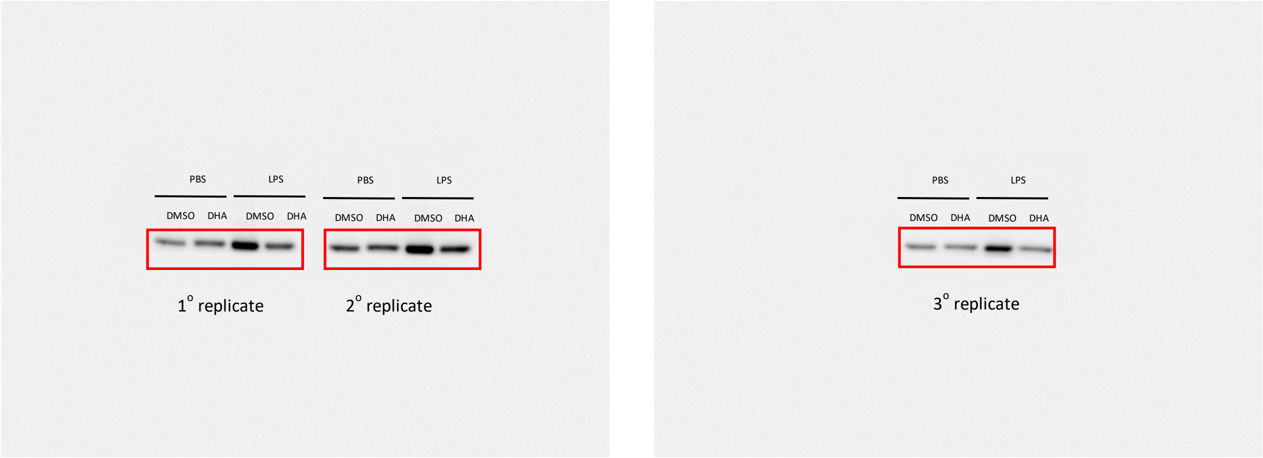

(Membrane was cut from middle)

T-STAT3 MW 79kDa

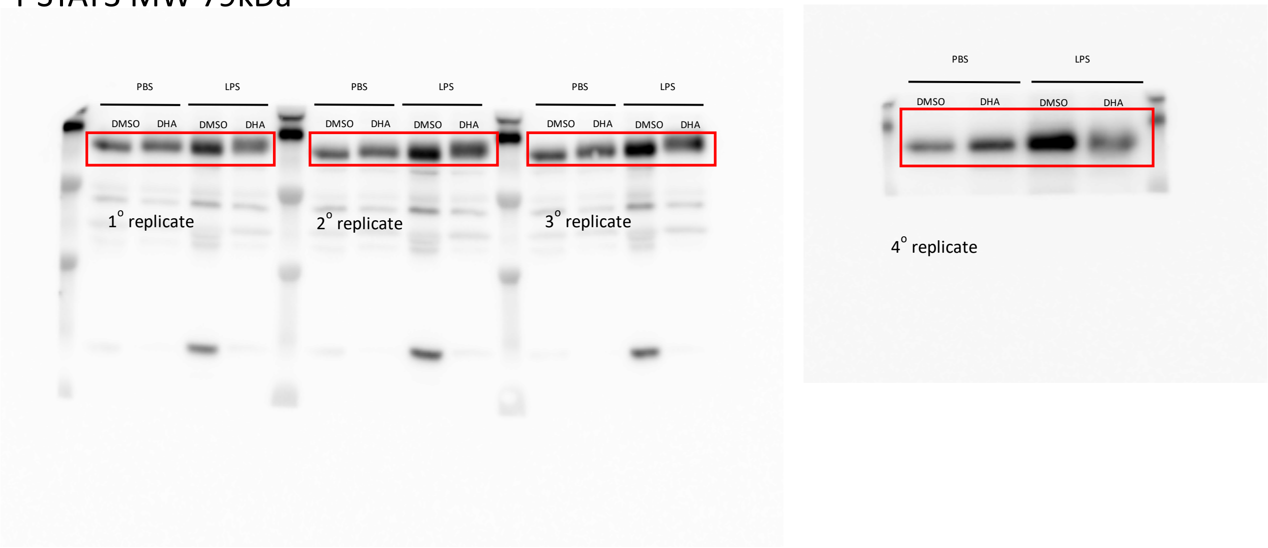

p70S6K MW 70kDa

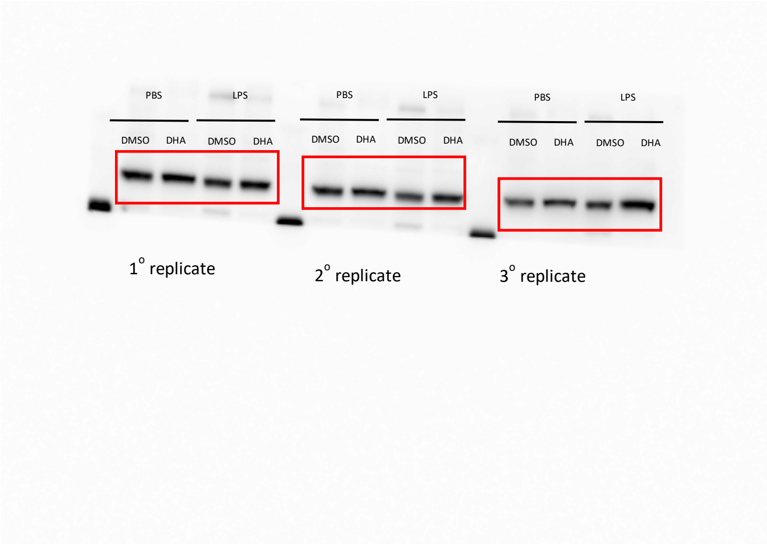

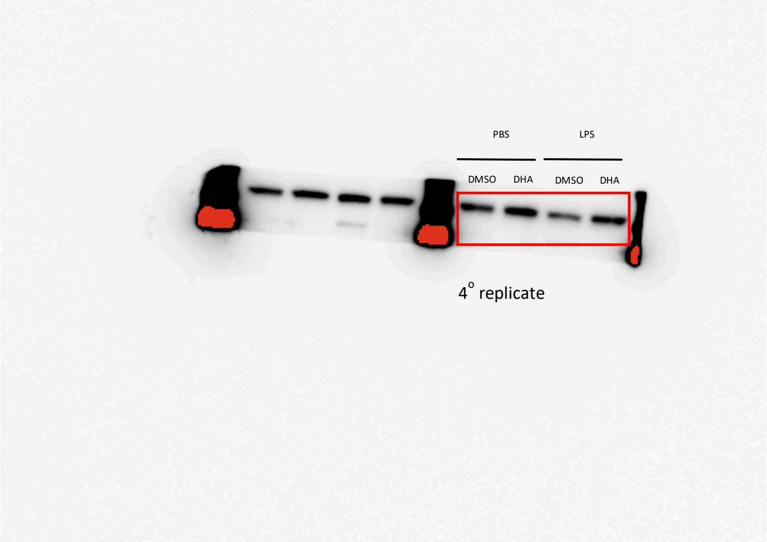

PGC1α/β MW 90kDa

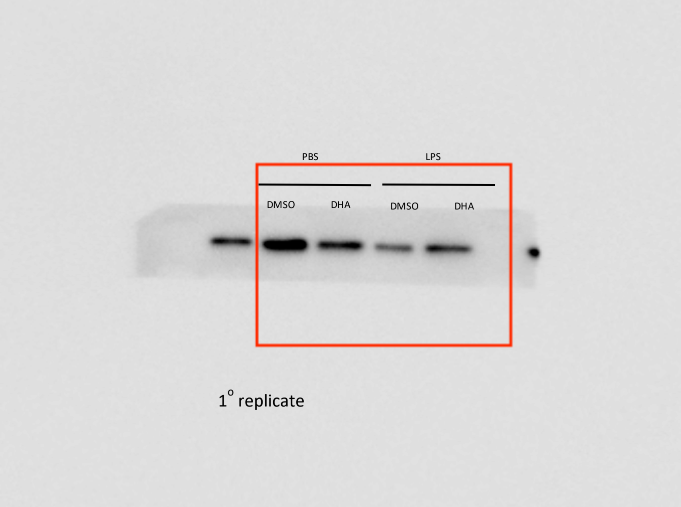

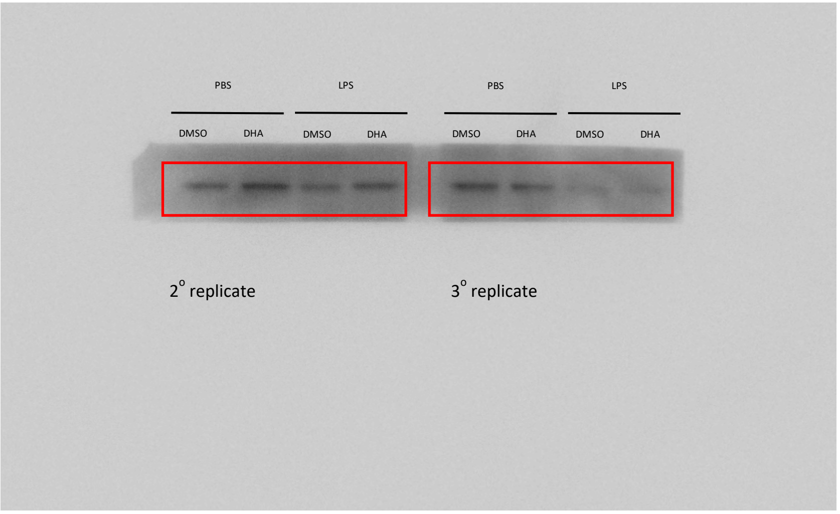

P-AKT MW 60kDa

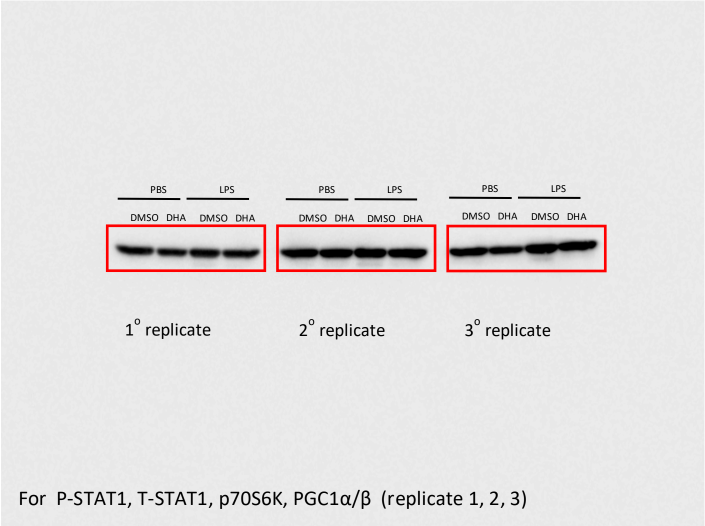

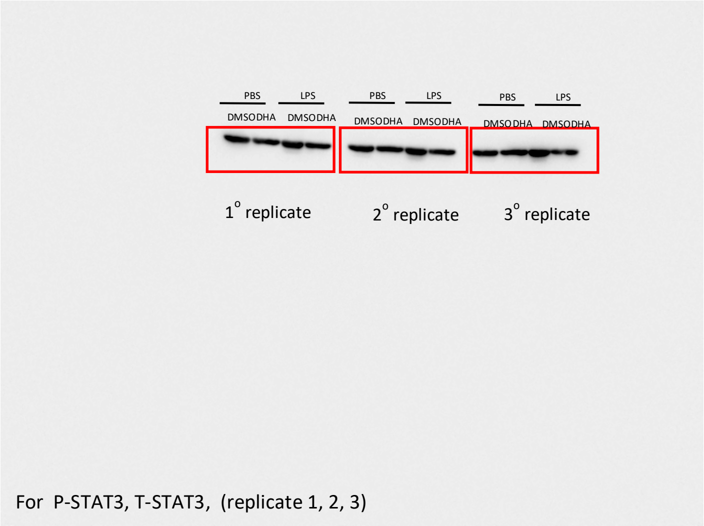

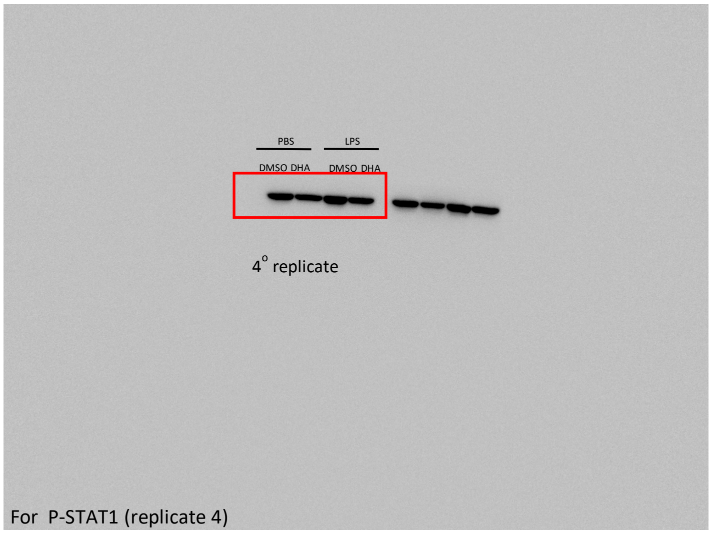

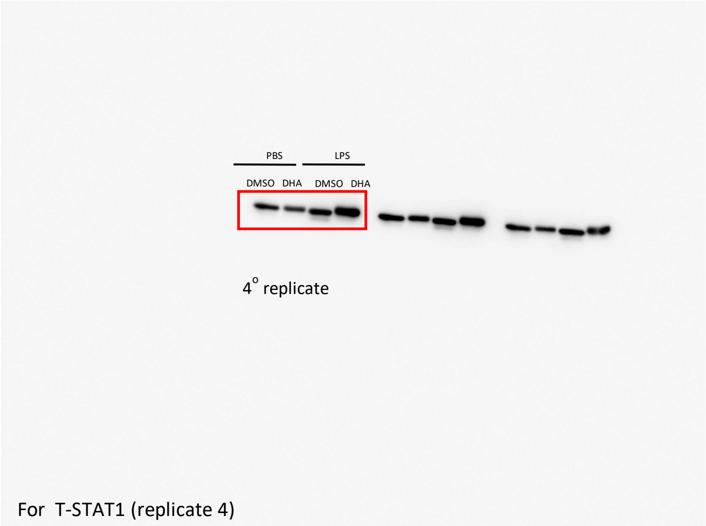

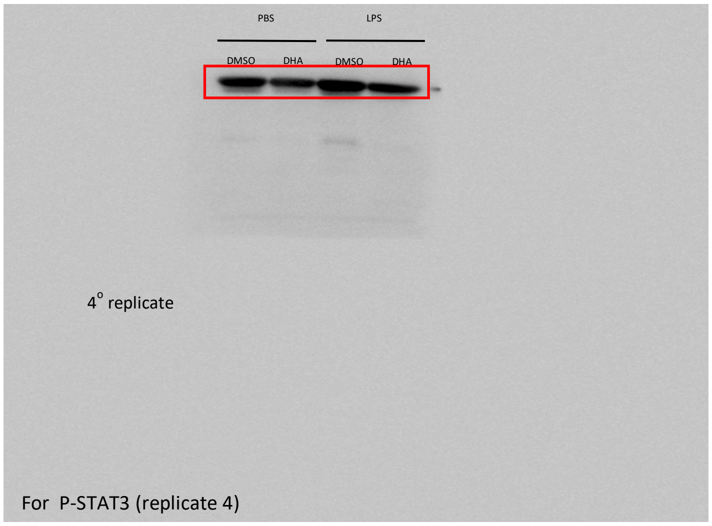

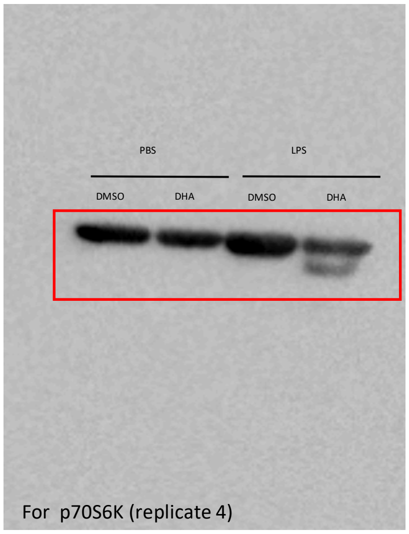

β-Actin MW 45kDa

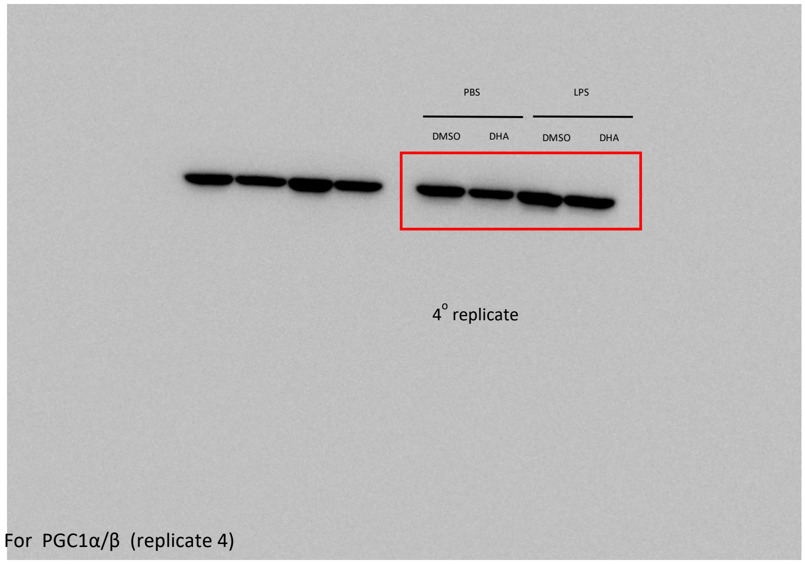

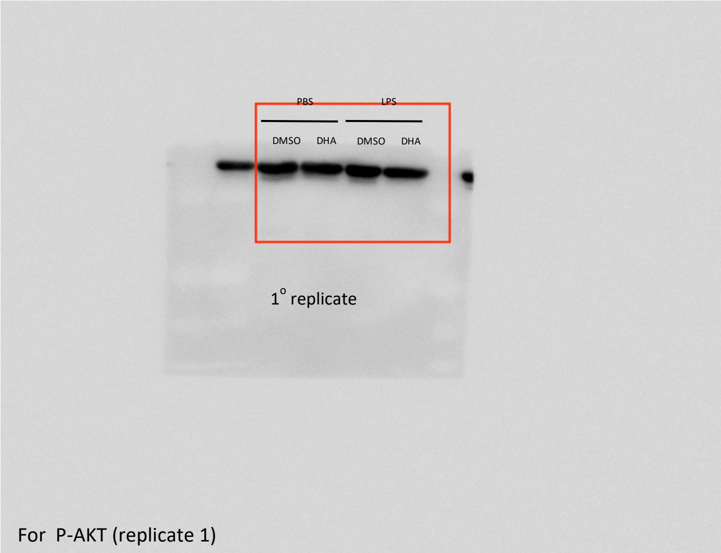

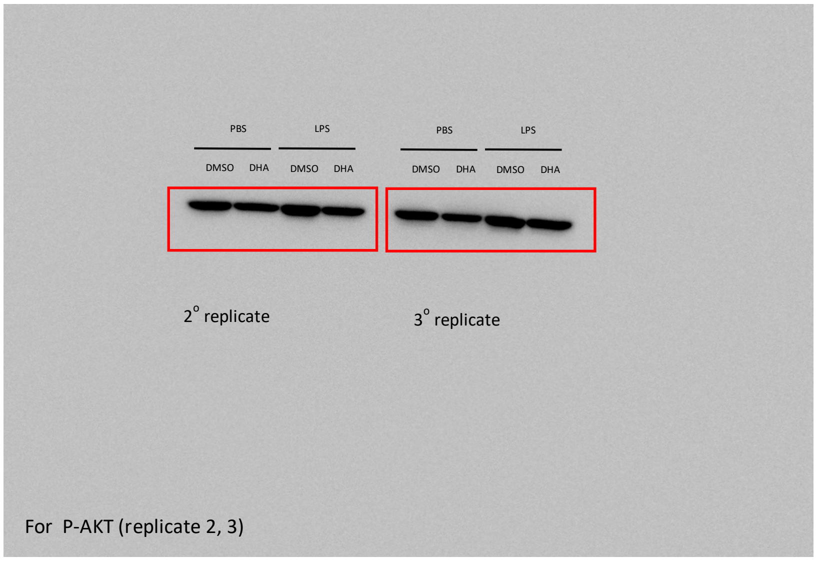

β-Actin MW 45kDa

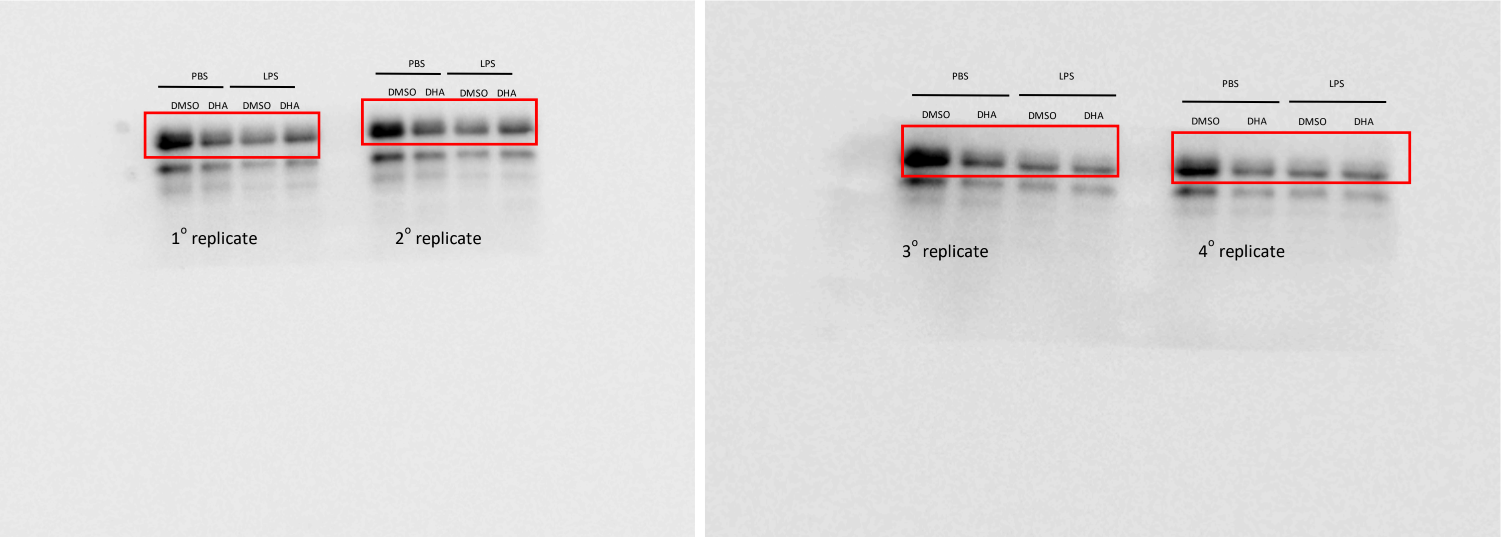

P-CREB MW 43kDa

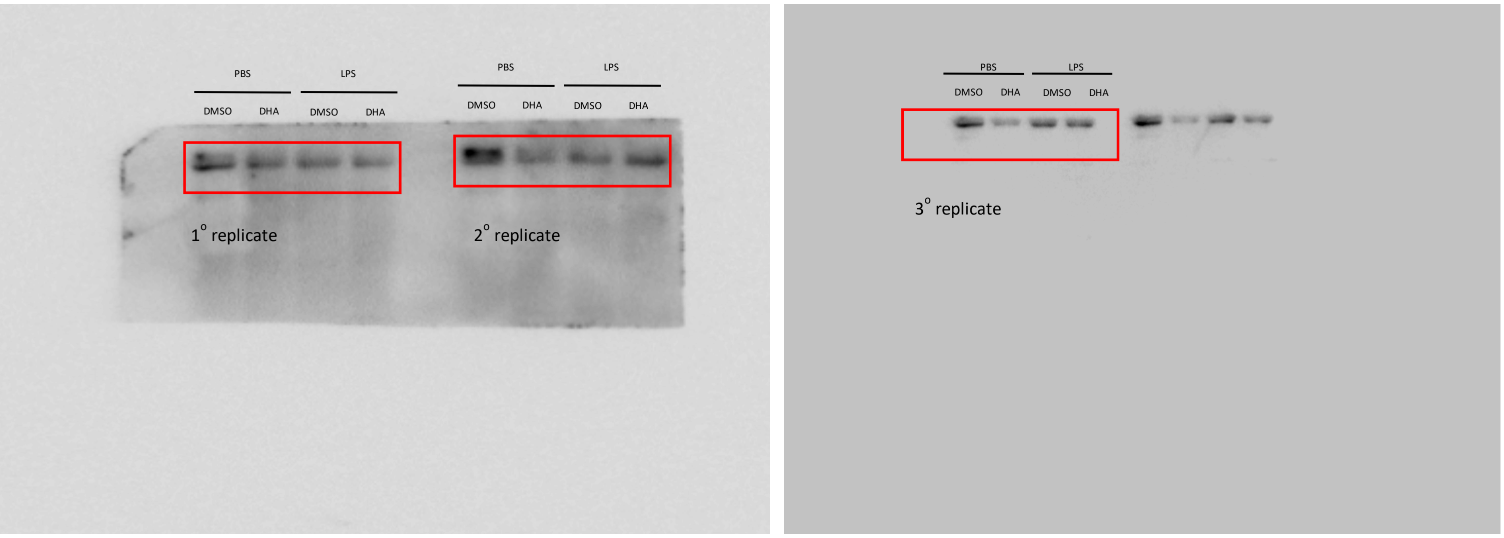

T-CREB MW 43kDa

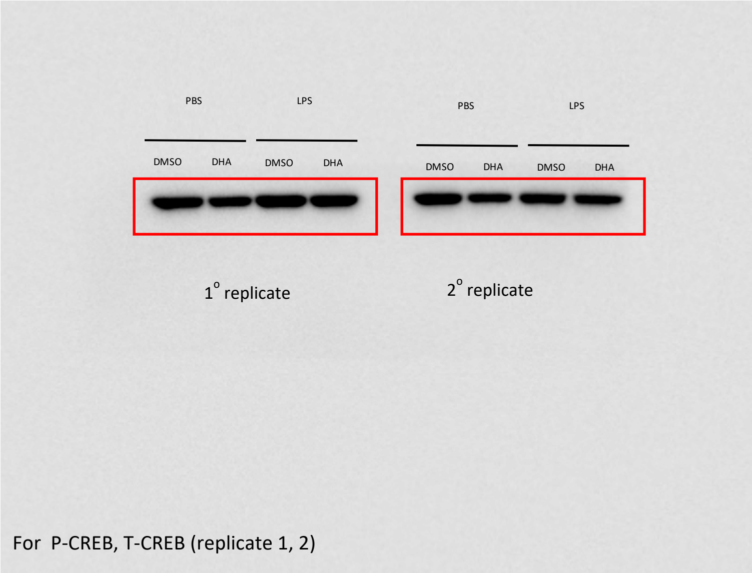

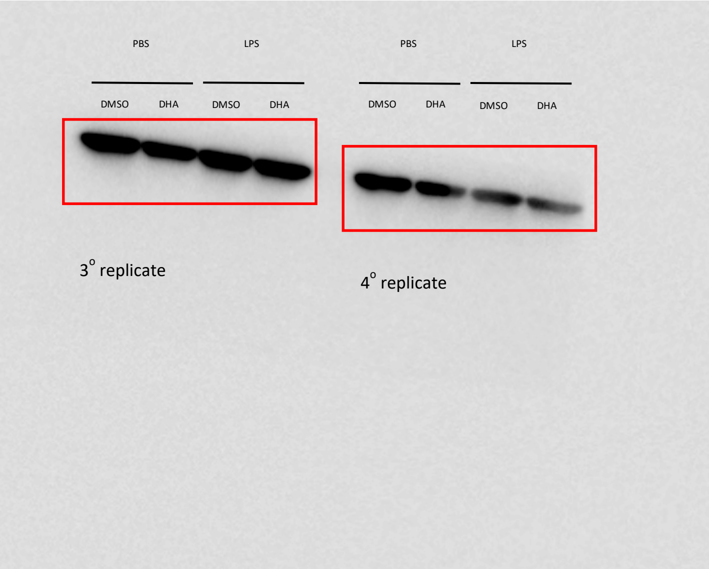

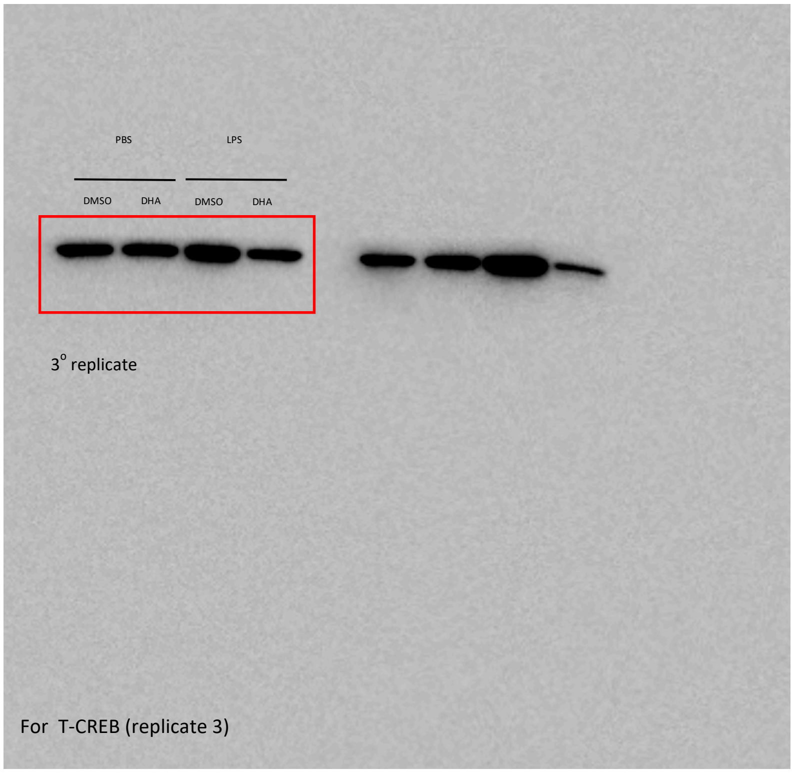

β-Actin MW 45kDa
